# A Pressure-Centered Mechanistic Framework for Precision Otology: The Neuro–Vascular–Mechanical–Inflammatory–Autonomic (NVMIA) Regulatory Architecture

**DOI:** 10.3390/jpm16060315

**Published:** 2026-06-12

**Authors:** Hee-Young Kim

**Affiliations:** 1 Department of Professional, Corporate, and Continuing Education, Harvard Medical School, Boston, MA 02115, USA; hee-young.kim-2022@ppcr.org; Tel.: +82-2-855-7541; 2 Center for Executive and Continuing Professional Education, Harvard T.H. Chan School of Public Health, Boston, MA 02115, USA; 3 Department of Otolaryngology, Kim Ear, Nose and Throat Clinic, Seoul 08753, Republic of Korea

**Keywords:** Eustachian tube dysfunction, middle ear pressure, pressure homeostasis, mechanistic framework, regulatory instability, precision medicine, systems physiology, barophysiologic regulation

## Abstract

Eustachian tube dysfunction (ETD) and related pressure-mediated otologic disorders often present with fluctuating auditory, vestibular, and pressure-related symptoms that are difficult to explain using static structural or symptom-based diagnostic labels alone. This conceptual review proposes the Neuro–Vascular–Mechanical–Inflammatory–Autonomic (NVMIA) framework as a hypothesis-generating architecture for organizing such variability. Within this framework, middle ear pressure (MEP) is interpreted as a clinically measurable physiologic variable through which interacting neural, vascular, mechanical, inflammatory, and autonomic influences may become mechanically expressed and clinically observable. The framework does not present NVMIA-based patterns as validated diagnostic categories, clinical decision tools, or treatment algorithms. Rather, it proposes provisional regulatory patterns that may help generate testable hypotheses regarding pressure-regulatory instability, cross-axis coupling, symptom fluctuation, and physiologic reversibility. Mechanical impedance may function as an accessible reference plane for future empirical assessment, while neural, vascular, inflammatory, and autonomic domains are conceptualized as modulatory axes that may alter symptom expression and response variability. The review further outlines future validation needs, including dynamic MEP measurement, patient-reported outcome integration, longitudinal response assessment, and cautious computational modeling. By reframing ETD as a model of state-dependent regulatory instability, the NVMIA framework provides a conceptual basis for future studies in precision otology while emphasizing that prospective validation is required before clinical implementation.

## 1. Introduction

### 1.1. Why Precision Medicine Requires Mechanistic Architecture

Precision medicine emerged from the recognition that patients who share the same diagnostic label often do not share the same underlying biology. Although structure- and symptom-based classifications remain clinically useful, they may be insufficient for disorders characterized by heterogeneity, temporal fluctuation, and multisystem interaction. In such conditions, diagnostic categories require physiologic organizing principles capable of linking biological regulation to measurable clinical expression.

Foundational precision medicine principles emphasize that disease should be understood as a network of interacting biological processes rather than as a static diagnostic entity [1]. Systems biology further emphasized nonlinearity, feedback organization, and emergent behavior [2], while physiological homeostasis depends on dynamically coupled regulatory systems rather than linear cause–effect chains [3]. Precision medicine therefore requires mechanistic architectures that identify variables governing system stability and provide testable links between regulation and clinical phenotype.

Eustachian tube dysfunction (ETD), characterized by impaired regulation of middle ear pressure (MEP), offers a clinically accessible model for examining such regulatory instability across auditory and vestibular domains.

### 1.2. Eustachian Tube Dysfunction as a Model of Regulatory Instability

Patients with ETD and related pressure-mediated disorders frequently present with fluctuating hearing loss, tinnitus, vertigo, aural fullness, and disequilibrium. These symptoms may coexist or shift over time, challenging attempts to localize pathology to a single structure or symptom domain.

Consistent with established ET physiology and international consensus definitions of ETD, MEP may be interpreted as more than a static parameter; rather, it represents a pressure-regulatory variable shaped by ET ventilation, mucosal dynamics, and reflexive control mechanisms [4,5]. Even modest pressure deviations can alter middle ear transmission through changes in impedance and mechanical loading [6,7,8], and may also influence vestibular symptom expression.

Historical and twentieth-century otologic observations further support the clinical association between impaired ET ventilation, pressure dysregulation, dizziness, tinnitus, aural fullness, fluctuating hearing disturbance, and vestibular–autonomic manifestations [9,10,11]. These observations do not validate a contemporary mechanistic framework, but they support the physiologic plausibility of considering pressure regulation as an organizing dimension in selected otologic presentations.

### 1.3. Aim of the Review

This conceptual review proposes a pressure-centered Neuro–Vascular–Mechanical–Inflammatory–Autonomic (NVMIA) framework for organizing ETD and related barophysiologic disorders. The framework conceptualizes pressure homeostasis as a dynamically regulated physiologic control system and describes how neural, vascular, mechanical, inflammatory, and autonomic domains may contribute to its instability.

The NVMIA framework is presented as a hypothesis-generating mechanistic architecture rather than as a validated diagnostic or therapeutic algorithm. Its purpose is to organize existing physiologic knowledge, identify testable regulatory relationships, and provide a structured basis for future empirical validation in precision otology.

## 2. Conceptual and Mechanistic Framework

### 2.1. Declarative Definition of the NVMIA Mechanistic Architecture

The Neuro–Vascular–Mechanical–Inflammatory–Autonomic (NVMIA) framework is proposed as a pressure-centered, hypothesis-generating architecture for organizing pressure-mediated otologic disorders. It integrates neural, vascular, mechanical, inflammatory, and autonomic processes within a reciprocally coupled regulatory system organized around middle ear pressure (MEP) as a clinically measurable physiologic variable.

Within this proposed architecture, MEP is not treated solely as a passive byproduct of isolated tubal dysfunction. Rather, it is conceptualized as a dynamically regulated coordinate through which distributed physiologic influences may become expressed as pressure-dependent mechanical states suitable for future empirical testing.

**Figure 1:**
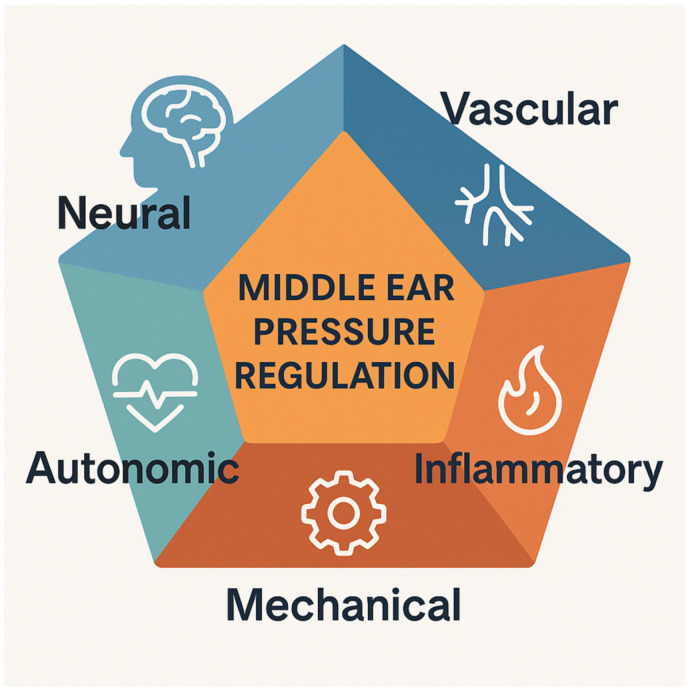
Pressure-centered regulatory architecture of the Neuro–Vascular–Mechanical–Inflammatory–Autonomic (NVMIA) framework. Middle ear pressure (MEP) is positioned as a clinically measurable physiological coordinate within a reciprocally coupled multiaxial regulatory system. Neural, vascular, mechanical, inflammatory, and autonomic domains are proposed to interact dynamically rather than function as isolated causal pathways. Pressure-mediated mechanical expression provides a shared interface through which heterogeneous regulatory perturbations may become clinically observable. The functional roles of each regulatory domain are summarized in Table 1.

The five domains are not arranged as a linear cascade or as independent causal entities. Instead, they are proposed to interact within a regulatory field of pressure homeostasis, where disturbances may arise in any domain and become clinically relevant through pressure-mediated mechanical expression. Within this conceptual model, precision otology is approached as the future identification and validation of pressure-mediated regulatory states, rather than as an already established clinical algorithm. The pressure-centered regulatory architecture of the proposed NVMIA framework is illustrated in Figure 1.

### 2.2. Pressure Homeostasis as a Regulatory System

#### 2.2.1. Pressure as a Dynamically Regulated Physiological Variable

MEP is conventionally treated as a passive mechanical consequence of ETD. Within the present framework, however, MEP is conceptualized as a dynamically regulated physiological variable that emerges from continuous interaction among ventilation, mucosal compliance, vascular tone, neural reflexes, and autonomic state.

Pressure equilibrium in the middle ear is not a static condition but a fluctuating regulatory target. Swallowing, yawning, posture, altitude variation, nasal airflow resistance, and autonomic tone all modulate the threshold and efficiency of ET opening. These adjustments occur within a closed-loop system in which pressure deviation triggers compensatory responses while simultaneously altering the mechanical boundary conditions of the auditory apparatus.

Thus, MEP homeostasis may be more appropriately understood as an active regulatory process rather than a binary state of “normal” or “abnormal.” In this context, instability may represent dysregulation of a pressure-control system rather than obstruction alone.

#### 2.2.2. Impedance Mechanics as the Clinically Observable Interface

The first clinically measurable expression of pressure regulation is often mechanical impedance. Variations in middle ear mechanical conditions, including pressure-related changes, may alter tympanic membrane compliance, ossicular loading, and energy transmission across the middle ear, producing quantifiable shifts in acoustic immittance and transfer characteristics [6,7,12].

Within the proposed NVMIA framework, these impedance states may be interpreted as clinician-readable outputs of distributed regulatory processes. Mechanical alteration does not necessarily imply that pathology originates in the mechanical axis; rather, it may reflect the integrated expression of neural coordination, mucosal condition, vascular modulation, and autonomic tone.

In this sense, mechanical impedance functions as a practical interface through which pressure-related regulatory instability may become measurable and suitable for future empirical assessment.

#### 2.2.3. Cochlear Boundary Coupling and Sensory Consequence

Pressure imbalance may influence auditory and vestibular physiology beyond conductive mechanics. Changes in MEP can reshape cochlear boundary conditions by altering oval- and round-window displacement mechanics, thereby modifying perilymphatic pressure gradients and influencing basilar membrane response patterns under susceptible conditions [8,13]. Recent mechanistic interpretations of ETD-related hearing disorders have emphasized that pressure dysregulation may influence conductive, sensorineural, and mixed auditory outcomes through mechanical and boundary-level pathways [14].

Even modest pressure deviations may alter sensory encoding fidelity by shifting the mechanical environment in which transduction occurs. Such changes could sensitize neural circuits, amplify perceptual gain, or lower thresholds for symptom generation, particularly when combined with inflammatory load, vascular vulnerability, or autonomic instability. These effects do not require primary cochlear degeneration, but they do require empirical clarification in longitudinal physiologic studies.

Within the NVMIA framework, pressure dysregulation is therefore proposed as a potential coupling mediator between middle ear mechanics and sensory perception. This perspective remains hypothesis-generating and requires prospective validation.

#### 2.2.4. Barophysiologic Stress and Threshold Crossing

When pressure instability is recurrent or sustained, the system may enter a state of barophysiologic stress. Repeated impedance fluctuation can increase mechanical and metabolic demand, alter microvascular resilience, and may engage vestibulo–autonomic reflex circuits. Symptom manifestation may occur when regulatory compensation is exceeded, even in the absence of progressive structural damage.

This threshold-crossing model may help explain why pressure-mediated disorders are frequently fluctuating, context-dependent, and potentially reversible. It also provides a plausible basis for understanding why similar structural findings may yield divergent clinical phenotypes depending on autonomic state, vascular reserve, inflammatory burden, or neural gain.

Within this proposed regulatory architecture, pressure homeostasis may operate as both transduction interface and a feedback mediator, anchoring multisystem regulation in mechanically expressed states. Future studies will be required to define the thresholds, temporal patterns, and response profiles that distinguish compensated from destabilized pressure-regulatory states.

### 2.3. Regulatory Architecture of the NVMIA Framework

Building on the conceptual definition above, the NVMIA framework organizes MEP regulation across five interacting domains: Neural, Vascular, Mechanical, Inflammatory, and Autonomic. These domains are proposed as functional dimensions of pressure-regulatory stability rather than independent etiologic categories.

Mechanical expression remains especially important because it provides an accessible measurable interface for pressure-related instability. However, the Mechanical domain is not presented as the universal origin of pathology; rather, it serves as a structural reference plane through which neural, vascular, inflammatory, and autonomic modulation may become clinically observable.

The following subsections summarize the proposed functional role of each domain. These descriptions are intended as conceptual constructs for future validation rather than as fixed diagnostic categories. The distinct regulatory contributions of each domain are summarized in Table 1.

**Table 1:** Functional roles of the Neuro–Vascular–Mechanical–Inflammatory–Autonomic (NVMIA) regulatory domains. This table summarizes the proposed functional contribution of each domain within the pressure-centered NVMIA architecture. The domains are intended as interacting regulatory dimensions rather than independent diagnostic categories.

Regulatory Domain	Functional Role
Neural	Sensory signal integration and central gain regulation
Vascular	Microvascular perfusion control and metabolic buffering mechanisms
Mechanical	Quantifiable biomechanical output and interface
Inflammatory	Mucosal biomechanical compliance and pressure-equilibration threshold modulation
Autonomic	State-dependent autonomic homeostatic control and reflex network integration

#### 2.3.1. Neural—Sensory Integration and Adaptive Gain Control

Within the proposed NVMIA framework, the Neural domain represents a regulatory interface through which pressure-related peripheral signals may be encoded, integrated, and adaptively modulated [15]. Neural pathways contribute to auditory and vestibular sensory processing while interacting with autonomic circuits that may influence physiologic susceptibility and symptom expression [16].

Alterations in MEP can modify afferent input from mechanically coupled middle and inner ear structures, thereby influencing sensory signals transmitted to brainstem nuclei and higher cortical networks. These signals are integrated with proprioceptive, autonomic, and contextual inputs to shape perception, reflex stabilization, and behavioral adaptation. Efferent neural pathways may also influence ET opening behavior, swallowing coordination, and middle ear muscle activity, contributing to pressure regulation [17,18].

Sustained or fluctuating pressure instability may promote adaptive changes within auditory and vestibular circuits, including modulation of neural gain and sensory weighting, even in the absence of progressive structural degeneration. Within the NVMIA architecture, the Neural domain is therefore proposed as a coupling domain through which pressure-mediated mechanical perturbations may influence sensory processing and central adaptive responses [15]. Susceptibility to motion-induced dizziness further supports the interaction between vestibular processing and autonomic responsiveness [16], while a representative case of ground-level alternobaric vertigo associated with ETD provides an illustrative clinical observation of pressure-related vestibular symptom coupling [19]. This observation is interpreted as hypothesis-generating rather than as validation of the NVMIA framework or a generalized therapeutic algorithm.

In some configurations, central adaptive processes may sustain symptom perception even after measurable mechanical parameters partially normalize. Such phenomena are interpreted as possible state-dependent amplification arising from prior or ongoing pressure-mediated perturbation, rather than as evidence of a purely neural etiology.

#### 2.3.2. Vascular—Microvascular Perfusion Regulation and Metabolic Resilience

Within the proposed NVMIA framework, the Vascular domain represents a modulatory dimension linking pressure dynamics to tissue resilience and recovery capacity. It is not presented as an isolated etiologic origin, but as a factor that may influence perfusion stability, metabolic reserve, and susceptibility to pressure-related perturbation.

Cochlear physiology depends on strial vascular integrity, which maintains endolymphatic homeostasis and the endocochlear potential [20,21]. Evidence that strial microcirculation is sensitive to systemic and local stressors supports the plausibility that vascular regulation may contribute to differences in sensory resilience under pressure-related stress [22].

In ETD-related contexts, recurrent MEP instability may impose abnormal mechanical loading on the tympanic membrane, ossicular chain, and cochlear windows. Although these effects are initially mechanical, sustained pressure disequilibrium may increase metabolic demand and challenge microvascular buffering capacity. This provides a plausible pathway through which pressure-mediated disorders may contribute to differences in auditory recovery and symptom persistence.

Vascular modulation may also influence mechanical expression indirectly. Microvascular tone and perfusion within peritubal and nasopharyngeal tissues may affect mucosal compliance, edema formation, and inflammatory susceptibility, thereby modifying the threshold at which pressure imbalance becomes clinically manifest. Autonomic–vascular interactions may further contribute to state-dependent variability in pressure regulation.

Within the NVMIA architecture, the Vascular domain is therefore proposed as a bidirectional modulatory domain linking pressure dynamics to tissue-level adaptability. Its role is hypothesis-generating and requires future studies examining whether vascular susceptibility modifies pressure-related symptom trajectories and recovery patterns.

#### 2.3.3. Mechanical—Structural Reference Plane and Quantifiable Impedance Interface

Within the proposed NVMIA framework, the Mechanical domain functions as the structural reference plane through which MEP regulation becomes clinically observable. It is not presented as the universal origin of pathology, but as the physical interface through which neural, vascular, inflammatory, and autonomic perturbations may be expressed as measurable impedance states.

Mechanical regulation of the middle ear depends on ET opening dynamics, middle ear compliance, ossicular loading, and pressure transmission across the tympanic membrane and cochlear windows. Alterations in these parameters may be detectable through established clinical tools such as tympanometry and wideband acoustic immittance [12,23], and negative MEP can produce measurable effects on auditory function [24].

Mechanical behavior should therefore be interpreted as an integrated output of upstream regulatory influences rather than as evidence of a singular mechanical cause. Inflammatory processes may increase mucosal stiffness or luminal resistance; autonomic imbalance may affect tubal opening coordination; neural modulation may influence middle ear reflexes; and vascular vulnerability may reduce tissue resilience to repetitive pressure stress. These influences can converge as impedance instability within a pressure-regulated system [8,25].

At a microstructural level, surface physics at the nasopharyngeal ET interface may also influence tubal opening. Opposed mucosal surfaces require transient separation during swallowing or reflexive maneuvers, and surface tension at the mucosa–air interface can influence the pressure threshold required for tubal opening [25,26]. Inflammatory exposure, mucosal secretion changes, or reflux-mediated irritation may therefore impair functional ventilation even in the absence of overt anatomical obstruction.

In this framework, the Mechanical domain provides a clinically accessible coordinate for future evaluation of pressure-regulatory instability. Its role is interpretive rather than causally exclusive, and its utility requires prospective validation through studies linking impedance measures, MEP dynamics, and patient-reported outcomes.

#### 2.3.4. Inflammatory—Mucosal Compliance Modulation and Pressure-Threshold Shifting

Within the proposed NVMIA framework, the inflammatory domain is conceptualized as a dynamic modulator of MEP regulation rather than as a fixed upstream cause or a purely secondary phenomenon. This interpretation is consistent with contemporary efforts to move ETD classification from broad clinical phenotypes toward more mechanism-oriented endotypes [27]. Inflammatory activity may alter mucosal compliance, epithelial surface properties, and neuromuscular coordination within the ET system, thereby modifying the energetic threshold and efficiency of pressure equalization without necessarily producing permanent structural obstruction.

Historical clinical observations suggested that upper airway disorders could influence middle ear symptoms by disrupting ET function [28]. Contemporary evidence further supports possible interactions between reflux-mediated mucosal irritation and pressure-regulatory dysfunction, in which inflammatory and mechanical instabilities may amplify one another within a coupled physiologic system [29,30].

From a biomechanical perspective, ET opening depends not only on muscular force but also on mucosal apposition, adhesion, viscoelastic tissue properties, and surface hydration. These factors are sensitive to inflammatory and mucosal surface conditions [4,25]. Increased surface adhesion and reduced mucosal compliance may elevate the energetic cost of tubal opening, destabilizing MEP homeostasis even in the absence of fixed anatomical obstruction.

Inflammatory modulation may also operate bidirectionally. Pressure instability may contribute to repetitive mechanical stress or altered airflow dynamics, while mucosal inflammation may impair ventilation and pressure equilibration. Reflux-related irritation provides one possible example of this pressure–inflammation coupling, but its role remains hypothesis-generating and requires prospective evaluation [29,30].

Within the NVMIA architecture, the inflammatory domain is therefore proposed as a modulatory dimension that may alter the stability, fluctuation pattern, and reversibility of pressure-mediated regulatory states. Its clinical relevance should be tested through future studies linking inflammatory markers, mucosal findings, MEP dynamics, and patient-reported symptom trajectories.

#### 2.3.5. Autonomic—State-Dependent Stability Regulation and Reflex Network Integration

Within the proposed NVMIA framework, the Autonomic domain represents a state-dependent regulatory dimension linking pressure dynamics to systemic physiologic context. Autonomic regulation may modulate vascular tone, mucosal perfusion, reflex excitability, and neuromuscular timing across the ET, middle ear, and vestibular systems.

Autonomic influence on pressure regulation may occur through several converging pathways. Sympathetic–parasympathetic balance can affect peritubal microvascular perfusion, mucosal compliance, surface hydration, and the threshold required for ET opening. These effects may dynamically alter the efficiency of pressure equalization, particularly under stress, fatigue, postural change, inflammatory burden, or systemic illness.

Autonomic regulation also interfaces bidirectionally with auditory and vestibular sensory processing. Disturbances in MEP may alter afferent input from mechanically coupled cochlear and vestibular structures, which interact with brainstem autonomic networks. Through vestibulo–autonomic coupling, pressure instability may contribute to nausea, vomiting, gastrointestinal discomfort, diaphoresis, or systemic unease in susceptible individuals [16,31]. Historical observations linking middle ear pressure imbalance with vestibular–autonomic symptoms further support the physiologic plausibility of this interaction [11].

Conversely, autonomic state may influence the capacity of the pressure-regulatory system to maintain stability under environmental or inflammatory stress. Such state-dependent variability may help explain why pressure-mediated symptoms fluctuate across contexts despite similar structural findings.

Within the NVMIA architecture, the Autonomic domain is therefore proposed as a modulatory dimension that may influence whether pressure perturbations remain compensated for or progress toward multisystem symptom expression. Its role remains hypothesis-generating and requires future studies linking autonomic markers, symptom timing, MEP dynamics, and longitudinal response patterns.

### 2.4. Functional Reference Ordering Within the NVMIA Framework

Within the proposed NVMIA framework, functional ordering does not imply a fixed causal hierarchy among regulatory domains. Rather, it refers to a provisional interpretive approach for considering which domain may be most influential in shaping pressure-regulatory instability at a given time.

The Mechanical domain occupies a distinctive position because MEP dynamics and impedance changes provide an accessible measurable interface through which distributed regulatory influences may become observable. However, this does not mean that mechanical dysfunction is the universal origin of pathology. Inflammatory, vascular, neural, and autonomic disturbances may each initiate or amplify pressure instability, with their effects often becoming clinically legible through pressure-dependent mechanical expression.

Experimental and theoretical studies demonstrate that changes in middle ear mechanical conditions can modulate impedance and acoustic energy transmission across the tympanic membrane and cochlear windows [6,7,23]. Such perturbations may influence cochlear input and vestibular perception [8,11]. The value of the Mechanical domain therefore lies in its role as a shared reference plane for interpretation, measurement, and future hypothesis testing rather than as a singular etiologic driver.

ET physiology further supports this reference ordering. ET opening behavior and pressure regulation are sensitive to mucosal state, muscular coordination, and surrounding physiologic context [4]. Accordingly, mechanical assessment may provide a practical coordinate system for studying heterogeneous upstream influences within a pressure-centered framework.

Table 2 summarizes this concept by linking each NVMIA domain to representative physiological roles, measurable features, and illustrative clinical expressions. The table is intended as a conceptual aid for future empirical testing rather than as a validated diagnostic checklist, clinical decision tool, or treatment guide.

### 2.5. Conceptual Integration: NVMIA as a Unified Physiologic Network

The NVMIA framework is proposed as a unified physiologic network rather than as a checklist of independent mechanisms. Its central premise is that pressure-mediated otologic symptoms may arise from dynamic coupling among neural, vascular, mechanical, inflammatory, and autonomic domains, with MEP serving as a clinically measurable regulatory coordinate.

Within this network, alterations in neural gain, vascular resilience, mucosal compliance, or autonomic tone may converge on ET opening dynamics and pressure regulation, producing measurable mechanical outputs [4,5,31]. Mechanical imbalance is therefore interpreted not as a universal primary cause, but as an accessible expression of the composite state of interacting regulatory systems.

Because these interactions are state-dependent, similar degrees of MEP deviation may be associated with different clinical expressions across individuals. Symptom severity and phenotypic pattern may depend not only on the magnitude of pressure deviation but also on the configuration of neural compensation, vascular reserve, inflammatory burden, and autonomic balance at a given time. This network perspective may help explain why ETD-related symptoms often fluctuate, reverse, or appear discordant with single-timepoint testing. Rather than treating variability as measurement error or functional overlay, the NVMIA framework interprets it as a potential signal of regulatory instability within a pressure-centered physiologic system. In this sense, phenotypic heterogeneity is interpreted not merely as diagnostic ambiguity, but as a possible expression of differing regulatory configurations across individuals and over time.

As a conceptual model, this network interpretation remains hypothesis-generating. Future studies will be needed to determine whether specific axis-coupling patterns can be measured reproducibly, predict symptom trajectories, and guide mechanism-oriented assessment and intervention studies.

### 2.6. Conceptual Transition Toward Future Precision Otology

The NVMIA framework positions ETD as a useful conceptual model for exploring precision-oriented approaches in otology because it centers on MEP, a measurable physiologic variable situated at the interface of multiple regulatory domains. Rather than conceptualizing ETD solely as a localized ventilation disorder, this framework interprets pressure regulation as a mechanical expression through which neural, vascular, inflammatory, and autonomic influences may become clinically interpretable [4,6,7].

This reconceptualization remains provisional. Its potential value lies in generating testable hypotheses regarding how measurable pressure-related variables, patient-reported symptom dynamics, and suspected regulatory-axis patterns may be integrated in future studies [32]. In this context, MEP and pressure-sensitive acoustic measures are considered candidate physiologic signals rather than validated surrogate biomarkers or treatment-directing indicators.

The framework may also provide a physiologically grounded representational structure for future computational modeling. However, such applications remain exploratory until MEP dynamics, axis-coupling patterns, and response trajectories are prospectively validated. In this sense, ETD may serve as a model domain through which principles of precision medicine can be explored within otology. By linking measurable regulation, multisystem interaction, and state-dependent symptom variability, the NVMIA framework provides a conceptual bridge linking pressure physiology with future mechanism-oriented assessment, computational interpretation, and validation studies.

Table 3 extends this transition by summarizing representative phenotypic patterns in relation to possible dominant and coupled NVMIA domains. Like Table 2, it is intended as a hypothesis-generating interpretive aid rather than a validated diagnostic classification, clinical decision tool, or treatment guide.

## 3. Middle Ear Pressure as a Candidate Surrogate Biomarker: A Conceptual Basis for Future Validation

MEP may be conceptualized as a physiologically grounded candidate surrogate biomarker within precision otology, because it reflects pressure-regulatory homeostasis and its functional coupling with auditory, vestibular, and autonomic systems. Within the NVMIA framework, MEP is interpreted not as a simple indicator of ventilation status but as a candidate integrative variable that may reflect clinically observable regulatory states. Its relevance derives from its proposed position within a coupled regulatory architecture rather than from isolated mechanical deviation alone.

In contemporary biomedical methodology, surrogate biomarkers are measurable variables intended to substitute for clinically meaningful endpoints and to predict therapeutic or pathophysiological outcomes [33]. However, designation as a surrogate biomarker requires more than physiologic plausibility or measurability. It requires analytical validity, clinical validity, responsiveness to intervention, and prospective evidence that changes in the biomarker reliably correspond to meaningful clinical outcomes.

Building upon prior conceptual proposals that aligned MEP with surrogate biomarker principles in ETD [32], the present framework provides a mechanistic context for structured evaluation. By situating MEP within a reciprocally coupled multiaxial regulatory network, this section outlines how pressure fluctuations may reflect integrated expressions of neural adaptation, vascular modulation, inflammatory threshold shifts, autonomic state variation, and mechanical boundary conditions.

Importantly, this conceptual positioning does not constitute empirical validation. Rather, it defines a physiologically coherent hypothesis space within which systematic validation studies can be designed. In this sense, MEP is not asserted as an established endpoint substitute but proposed as a mechanistically justified candidate surrogate whose validation trajectory may become methodologically tractable within a pressure-centered precision model.

### 3.1. Interpreting MEP as an Index of Baroregulatory Stability

The middle ear is a pressure-sensitive biomechanical chamber that is anatomically and functionally coupled to the nasopharynx, cochlea, and vestibular labyrinth. Variations in MEP can alter tympanic membrane compliance, ossicular chain loading, and the boundary conditions at the oval and round windows, thereby modifying acoustic impedance and mechanical energy transmission across the middle ear system [6,7].

However, the physiological significance of MEP extends beyond sound conduction mechanics. Because pressure gradients define part of the mechanical interface between the external environment and the inner ear, deviations in MEP may reshape cochlear input impedance and influence labyrinthine fluid displacement dynamics under susceptible conditions. These changes may affect neural encoding, central gain regulation, and vestibular responsiveness, particularly when pressure states are asymmetric or rapidly fluctuating [16,31].

MEP should therefore not be interpreted as an isolated mechanical variable. Rather, within the proposed NVMIA framework, it is considered a clinically accessible readout of interacting regulatory domains. Vascular microperfusion may influence tissue resilience under pressure stress; inflammatory activity may alter mucosal compliance and surface mechanics at the ET interface; autonomic tone may modulate tubal opening efficiency and temporal pressure equilibration; and neural reflex pathways may coordinate adaptive compensation. The measured pressure state may therefore reflect the net expression of interacting regulatory influences rather than a singular mechanical defect.

Within this framework, MEP may serve as an index of baroregulatory stability, capturing the dynamic relationship between environmental pressure demands and physiologic regulatory capacity. Similar absolute pressure values may produce different clinical consequences depending on axis configuration and proximity to compensatory thresholds. This state-dependent variability supports the potential value of MEP as a candidate surrogate biomarker, while underscoring the need for prospective validation linking MEP dynamics to clinically meaningful outcomes.

### 3.2. MEP Dysregulation as a Mechanistic Trigger

When baroregulatory stability is compromised, deviations in MEP may act as perturbation signals within the middle ear and inner-ear mechanical environment. Negative or unstable MEP can increase stiffness of the tympanic membrane–ossicular system, alter stapes footplate excursion, and modify impedance at the oval and round windows, thereby reshaping cochlear fluid–pressure gradients [6,7]. Clinically, these mechanical changes may contribute to low-frequency conductive attenuation characterized by a stiffness-dominated audiometric configuration, fluctuating thresholds, aural fullness, and pressure intolerance [24].

The pathophysiologic impact of MEP dysregulation may extend beyond conductive mechanics. Altered pressure boundary conditions influence cochlear input impedance and may modulate neural encoding within the auditory pathway, contributing to tinnitus fluctuation, sound sensitivity, and perceptual instability in susceptible individuals. In parallel, asymmetric or rapidly changing pressure states can perturb vestibular loading, particularly through altered stapes–vestibular coupling, providing a plausible substrate for pressure-sensitive dizziness and non-rotatory vertigo [6,31].

The triggering potential of MEP dysregulation may be most clinically relevant during dynamic barometric or physiologic transitions rather than during static pressure states. Swallowing, yawning, head movement, altitude change, or rapid environmental pressure shifts transiently amplify trans-tympanic and labyrinthine gradients. When regulatory buffering capacity is reduced, these dynamic pressure excursions may exceed compensatory thresholds and engage neural or autonomic responses via vestibulo–autonomic reflex pathways. Clinical manifestations may include disequilibrium, nausea, diaphoresis, dyspnea, palpitations, or anxiety-like sensations, although these associations require further prospective characterization.

Within the NVMIA framework, MEP dysregulation is therefore proposed not as an isolated mechanical abnormality, but as a perturbation node within a coupled regulatory network. The magnitude of symptom expression may depend not only on absolute pressure values, but also on interactions among pressure deviation, neural gain, vascular resilience, inflammatory modulation of mucosal compliance, and autonomic balance. This interaction provides a plausible explanation for why similar measured pressure states may provoke minimal symptoms in one context yet more severe multisystem manifestations in another.

### 3.3. ET Opening Mechanics and MEP Homeostasis

The ET functions as the dynamic valve governing MEP homeostasis. Effective pressure regulation depends on coordinated muscular activation—primarily mediated by the tensor veli palatini—together with sufficient mucosal compliance, intact neuromuscular timing, and a permissive inflammatory and autonomic milieu [4,17]. Disruption at any level of this coordinated system may compromise pressure equalization despite preserved gross anatomy.

ETD therefore does not require complete luminal obstruction. Subtle abnormalities—including delayed opening, incomplete dilation, reduced mucosal compliance, or impaired neuromuscular synchronization—may sustain recurrent cycles of negative pressure and barophysiologic instability. Such functional disturbances may contribute to chronic or fluctuating MEP deviation even when imaging and endoscopic findings appear structurally unremarkable [5,17].

Inflammatory modulation may further alter opening mechanics by increasing mucosal edema, surface adhesion, and tissue stiffness at the nasopharyngeal ET orifice. Conditions such as allergic rhinitis or laryngopharyngeal reflux may transiently elevate the energetic threshold required for tubal opening, thereby amplifying pressure instability in a state-dependent manner [4,5]. These effects are dynamic rather than fixed and may fluctuate with inflammatory burden, mucosal surface conditions, and autonomic tone.

Within this mechanistic context, measured MEP may reflect the downstream equilibrium—or disequilibrium—of ET regulatory performance. Persistent or unstable negative pressure may indicate impaired neuromuscular coordination, inflammatory vulnerability, autonomic imbalance, or combined regulatory strain rather than a singular structural defect. MEP is therefore interpreted within the NVMIA framework as a candidate integrative readout of ET system performance across interacting regulatory domains.

In precision otology, interpretation of pressure measurement should extend beyond static ventilation status. Longitudinal assessment of MEP may provide a clinically accessible approach to evaluating pressure-homeostatic control, linking ET opening mechanics to broader multisystem physiology and helping explain the temporal variability, context sensitivity, and reversibility characteristic of functional ETD.

### 3.4. Limitations of Current Clinical Measurement

Conventional assessment of MEP relies predominantly on single-timepoint tympanometry, which captures only a static representation of a fundamentally dynamic regulatory process. Although tympanometry remains a valuable screening and monitoring tool, its cross-sectional nature limits its ability to characterize temporal variability, context sensitivity, and state-dependent fluctuations inherent to pressure-mediated disorders [4].

Static measurements do not fully resolve pressure dynamics occurring during physiologic events such as swallowing, yawning, phonation, posture change, or transient barometric shifts. Consequently, individuals with intermittent or context-dependent ETD may demonstrate normal tympanometric findings despite clinically meaningful symptom burden—a limitation recognized in contemporary consensus definitions of ETD [5].

Furthermore, isolated pressure values do not identify the upstream modulatory influences that shape baroregulatory stability. Autonomic modulation of tubal mechanics, inflammatory variation in mucosal compliance, and vascular contributions to cochlear resilience cannot be inferred reliably from single MEP measurement. Although cochlear homeostasis and micropressure regulation have been extensively studied [21], their interaction with dynamic MEP states remains insufficiently characterized in routine clinical practice.

These limitations underscore why MEP should not be treated as a validated surrogate biomarker on the basis of single-point measurement alone. Instead, its potential value lies in dynamic and longitudinal assessment, including repeated pressure measurements, interaural asymmetry patterns, symptom-linked variability, and response trajectories following pressure-related clinical intervention.

Within the proposed precision otology paradigm, future measurement strategies should aim to capture MEP as a dynamic, context-sensitive regulatory signal. Longitudinal pressure assessment, integration with physiologic state variables, and alignment with patient-reported outcomes may help define whether MEP dynamics can serve as a reliable candidate surrogate marker of baroregulatory stability.

Computational modeling approaches, including AI-assisted temporal pattern recognition, may eventually support quantification of variability, cross-axis coupling, and regulatory thresholds across NVMIA domains. However, such applications remain dependent on prospective validation of physiologic inputs and clinically meaningful outcomes.

By transitioning from static snapshots to dynamic, mechanism-aware analysis, precision otology may reconceptualize MEP not merely as an isolated measurement, but as a candidate system-level signal through which multisystem regulation—and dysregulation—can be studied.

### 3.5. MEP as the Central Node of NVMIA Integration

Within the NVMIA framework, MEP occupies a proposed central position as a clinically accessible convergence point through which interacting regulatory domains may become observable. It represents a clinically measurable interface through which neural, vascular, inflammatory, mechanical, and autonomic variability becomes translated into pressure-mediated phenotypic expression.

Neurally, fluctuations in MEP may reshape afferent input patterns, influencing central gain regulation, tinnitus modulation, and vestibular sensitivity [15,16]. Vascular contributions interact with pressure stability by modulating cochlear microenvironmental resilience and metabolic reserve, thereby affecting susceptibility to reversible versus persistent dysfunction [21]. Inflammatory processes may alter mucosal compliance and surface mechanics at the ET interface, lowering the threshold at which pressure disequilibrium becomes symptomatic [4,5]. Mechanically, MEP influences tympanic membrane stiffness, ossicular mobility, and oval- and round- window boundary conditions, producing measurable effects on conductive thresholds and pressure intolerance [6,7,24]. Autonomically, pressure instability may engage vestibulo–autonomic reflex circuits and stress-modulated tubal coordination, contributing to systemic manifestations such as nausea, disequilibrium, and pressure-sensitive dizziness in susceptible individuals [31].

Positioning MEP at this intersection may clarify why diverse symptom constellations—including fluctuating hearing loss, tinnitus, vertigo, aural fullness, reflux-associated manifestations, and swallowing-induced dizziness—can arise from distinct mechanistic configurations while sharing a common pressure-regulatory substrate. It may also help generate hypotheses regarding why changes in pressure homeostasis may be associated with multisystem symptom changes in some patients despite limited structural findings.

Thus, MEP may function as a candidate systems-level integrative signal within the NVMIA network. Its proposed centrality provides the conceptual bridge between measurable physiology and heterogeneous clinical presentation, forming a basis for future mechanism-guided phenotyping and validation studies capable of capturing dynamic, state-dependent disease expression.

### 3.6. Mechanistic Conclusions

Within the proposed NVMIA framework, MEP is best conceptualized as a candidate integrative physiologic signal that may reflect coordinated regulation across neural, vascular, mechanical, inflammatory, and autonomic domains. Its instability should not be interpreted solely as a local mechanical disturbance but may represent a measurable expression of multiaxial regulatory disequilibrium.

By translating upstream physiological variability into pressure-dependent mechanical states and symptom patterns, MEP may provide a unifying conceptual substrate for interpreting the heterogeneous and fluctuating manifestations of ETD. This perspective may help reconcile discordant audiologic, vestibular, and patient-reported findings by situating them within a pressure-centered regulatory architecture.

However, MEP should not yet be regarded as a validated surrogate biomarker or established endpoint substitute. Its current value lies in defining a mechanistically coherent and testable hypothesis space for future validation studies. Such studies should determine whether dynamic MEP measures, interaural pressure asymmetry, longitudinal variability, and response to pressure-stabilizing or physiology-oriented approaches predict clinically meaningful outcomes.

Centering diagnostic reasoning on a measurable and potentially reversible regulatory variable may help reframe ETD as a mechanistically tractable systems-level condition. In doing so, the NVMIA framework provides a conceptual and translational foundation for future mechanism-guided precision otology through longitudinal monitoring, mechanism-informed intervention, and computational modeling of pressure-regulatory dynamics.

## 4. Diagnostic Pathways for Precision Otology

Recognition of MEP as a dynamic, pressure-regulatory signal requires a corresponding shift in diagnostic reasoning. Within the proposed NVMIA framework, diagnostic interpretation is not intended to replace conventional ETD evaluation or to function as a validated clinical algorithm. Rather, it is presented as a conceptual pathway for organizing temporal variability, contextual modulation, and multisystem coupling around measurable pressure-mediated physiology.

Historically, evaluation of ETD has depended on cross-sectional assessments, including categorical tympanometry and symptom questionnaires [4]. While clinically useful, such approaches often fail to detect intermittent, baro-challenge-dependent, or autonomically modulated dysfunction [5]. When dynamic instability rather than fixed structural abnormality predominates, pressure-mediated disorders may be misclassified as functional or irreversible inner ear disease.

A precision-oriented diagnostic pathway therefore emphasizes dynamic physiology. Objective pressure-sensitive measures should be interpreted alongside longitudinal symptom patterns and contextual triggers, consistent with mechanism-based precision medicine principles that emphasize state dependence and network-level interpretation over static labels [1].

Accordingly, this section outlines a conceptual multimodal diagnostic architecture aligned with the NVMIA framework. Its purpose is to map available clinical tools to proposed regulatory domains and to identify how MEP-centered assessment may support future mechanism-oriented phenotyping, while emphasizing that prospective validation remains necessary.

### 4.1. Patient-Reported Outcomes (PROMs): Capturing Variability and Context Dependence

Patient-reported outcomes (PROMs) may provide an early clinical signal of pressure-regulatory instability by capturing symptoms that fluctuate across time, context, posture, swallowing, barometric exposure, inflammatory burden, and autonomic state. In ETD and related pressure-mediated disorders, symptoms such as aural fullness, tinnitus, fluctuating hearing, dizziness, pressure intolerance, throat discomfort, and reflux-associated sensations often vary in ways that are not fully captured by single-timepoint physiological testing.

Within the proposed NVMIA framework, PROMs are not treated as diagnostic substitutes for objective assessment. Rather, they are interpreted as patient-centered phenotypic signals that may help identify temporal patterns, contextual triggers, and multisystem symptom clustering. Such information may guide selection of confirmatory physiological tests and inform hypotheses regarding dominant or coupled regulatory axes.

Existing ETD-specific PROMs, including the ETDQ-7, provide valuable symptom quantification and may assist in assessing ETD-related symptom burden [34,35]. However, current PROMs do not fully capture the broader pressure-mediated symptom spectrum, particularly vestibular, autonomic, reflux-associated, interaural, and context-dependent manifestations. This limitation highlights the need for future PROM modules that integrate pressure sensitivity, symptom fluctuation, interaural perception, swallowing-related changes, and barometric responsiveness.

PROMs may therefore contribute to precision otology by linking subjective symptom experience to measurable physiological dynamics. However, their role within the NVMIA framework remains hypothesis-generating and must be validated against objective measures such as tympanometry, wideband acoustic immittance, longitudinal MEP patterns, vestibular testing, and treatment-response profiles.

### 4.2. Conventional Objective Diagnostics: Anchoring the Baseline

Pure-tone audiometry, impedance testing, and tympanometry remain foundational components of ETD evaluation [4,36]. These tools document conductive effects, stiffness-related changes, effusion, and baseline ventilation status, providing essential exclusionary information and reference metrics.

However, they capture only instantaneous mechanical states. Static tympanometric classification may fail to reveal intermittent or state-dependent MEP instability. Similarly, audiometric fluctuation documents downstream consequence but does not distinguish reversible mechanical loading from vascular or regulatory perturbation [21].

Within precision otology, conventional diagnostics anchor interpretation but do not fully define pressure-regulatory state. They establish the structural and mechanical baseline against which dynamic instability can be interpreted. Additional assessment of temporal variability, symptom-linked pressure fluctuation, interaural asymmetry, and response to physiologic maneuvers may help characterize baroregulatory instability more accurately than single-timepoint classification alone.

These approaches are not intended to function as validated diagnostic algorithms at this stage, but rather as candidate measurement strategies for future mechanism-oriented phenotyping. Prospective studies are needed to determine whether dynamic MEP patterns, interaural asymmetry, and pressure-response profiles predict patient-reported outcomes, vestibular symptoms, auditory fluctuation, or response trajectories following pressure-related clinical intervention.

### 4.3. Dynamic and Pressure-Sensitive Modalities: Rendering Regulation Measurable

Advanced pressure-sensitive modalities may help evaluate MEP regulation under physiologic challenge, shifting emphasis from static structural assessment toward dynamic control. These approaches are particularly relevant when symptoms are intermittent, context-dependent, or discordant with resting tympanometry.

Wideband acoustic immittance (WAI) quantifies frequency-specific absorbance across pressure spectra and may detect pressure-sensitive mechanical perturbations that are not fully captured by standard tympanometry [37,38]. Sonotubometry and tubomanometry assess ventilatory competence and ET opening thresholds during challenge conditions, potentially revealing intermittent dysfunction that resting measures may miss [17]. Endoscopic visualization adds structural–functional correlation by helping distinguish fixed obstruction from dynamic mucosal or functional dysregulation.

Within the proposed NVMIA framework, these tools may be interpreted as complementary measurement layers rather than direct diagnostic markers of individual axes. WAI primarily informs the Mechanical domain by characterizing impedance-related perturbation; mucosal and endoscopic findings may support interpretation of Inflammatory modulation; ventilatory variability during challenge may reflect mechanical opening efficiency influenced by inflammatory, autonomic, or neuromuscular factors; and downstream auditory or vestibular consequences may raise hypotheses regarding Neural or Vascular coupling.

Dynamic diagnostics may therefore help move MEP assessment from isolated static measurement toward a more contextualized regulatory signal. However, axis assignment based on these modalities remains provisional and requires prospective validation against longitudinal pressure profiles, symptom trajectories, and treatment-response patterns.

**Figure 2:**
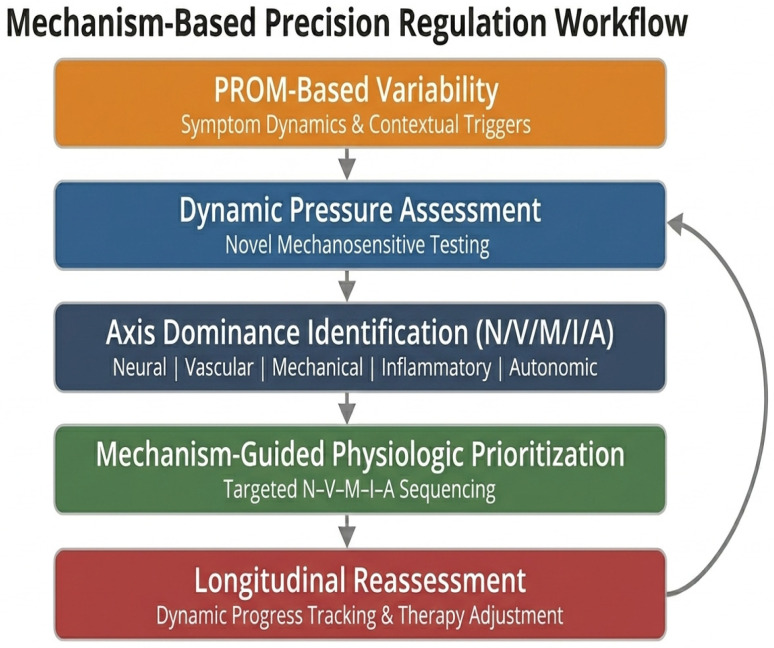
Conceptual diagnostic pathway for pressure-centered precision otology within the Neuro–Vascular–Mechanical–Inflammatory–Autonomic (NVMIA) framework. The figure illustrates how conventional diagnostics, pressure-sensitive dynamic testing, patient-reported outcomes, and contextual clinical features may be integrated to generate provisional hypotheses regarding dominant or coupled NVMIA regulatory domains. This pathway is intended as a conceptual interpretive model rather than a validated diagnostic algorithm. Axis assignment remains hypothesis-generating and should be tested prospectively against longitudinal MEP profiles, symptom trajectories, and treatment-response patterns.

The structured integration of these measurement layers is summarized in Figure 2.

### 4.4. Multimodal Integration: Scaling Mechanistic Interpretation

AI applications in otolaryngology have expanded rapidly in recent years. Machine learning systems have been developed for the differential diagnosis of vertigo and dizziness [39], deep learning-based audiogram classification [40], and broader audiologic applications summarized in contemporary scoping reviews [41]. Smartphone-based videonystagmography platforms further demonstrate that AI-enabled ocular motor analysis can be deployed at scale [42], while large clinical datasets have supported decision-support models for vestibular disorder classification [43]. At the implementation level, however, translational commentary continues to highlight workflow, interpretability, and regulatory challenges in routine Ear, Nose and Throat practice [44].

These advances reflect substantial progress in pattern recognition. However, many current diagnostic architectures remain primarily trained on downstream phenotypic outputs, including eye movements, sway metrics, audiometric configurations, imaging features, or symptom clusters. In pressure-mediated otologic disorders, such outputs may not fully capture the regulatory variables governing system stability.

In barophysiologic phenotypes, this limitation may be clinically relevant. MEP variability can modulate afferent signaling, alter cochlear impedance, and influence vestibular integration without necessarily producing fixed structural abnormalities. When MEP dynamics are absent from the model’s feature space, AI systems may classify observable patterns while incompletely representing upstream pressure-regulatory mechanisms. Algorithmic performance should therefore not be interpreted as equivalent to physiological representation.

Within precision otology, the NVMIA framework may provide a structured physiologic scaffold for future mechanism-aware computational modeling. Rather than positioning AI as an autonomous diagnostic authority, this approach suggests that computational systems should be guided by clinically meaningful physiological variables, including dynamic MEP patterns, impedance modulation, inflammatory burden, vascular susceptibility, neural adaptation, and autonomic state markers.

In this configuration, AI would not replace physiologic reasoning but may help organize and test it at scale. However, applications such as axis-dominance detection, dynamic coupling analysis, or treatment-response prediction remain future objectives requiring prospective validation. The immediate value of the NVMIA framework is therefore not to establish an immediately deployable AI diagnostic framework, but to identify physiologic variables that should be considered in the design of interpretable, mechanism-aware models.

### 4.5. Diagnostic Conclusions

A precision diagnostic pathway in otology may require a shift from static classification toward dynamic, physiology-centered assessment. Within the proposed NVMIA framework, diagnosis is not reframed as a replacement for conventional evaluation, but as a conceptual process for interpreting isolated test findings in relation to pressure-centered regulatory imbalance and clinical expression.

By integrating PROMs, conventional objective measures, pressure-sensitive modalities, and future multimodal computational approaches, MEP may be positioned as a candidate systems-level regulatory signal rather than a secondary mechanical variable alone. This reorientation may provide a basis for future mechanism-oriented phenotyping, provided that dynamic pressure profiles, axis-coupling patterns, and treatment-response relationships are prospectively validated.

More fundamentally, this diagnostic perspective interprets otologic variability not simply as diagnostic ambiguity, but as a potential expression of measurable regulatory instability. In this way, pressure homeostasis may serve as a clinically interpretable reference framework for precision otologic reasoning and as a conceptual bridge toward the mechanism-oriented phenotyping strategy discussed in the following section.

## 5. Illustrative NVMIA-Based Phenotypic Patterns in Barophysiologic Disorders

Building upon the precision diagnostic pathway outlined in Section 4, this section presents illustrative phenotypic patterns within the NVMIA framework. These patterns are intended to show how dynamic pressure-related signals may be conceptually organized according to suspected regulatory predominance, cross-axis coupling, and potential reversibility of regulatory patterns, rather than to establish a validated clinical classification system.

Experimental and clinical studies suggest that alterations in MEP, even in the absence of effusion, can produce measurable mechanical changes along the auditory transmission chain. Pressure shifts may modify tympanic membrane stiffness and ossicular loading, alter cochlear boundary mechanics at the oval and round windows, and influence cochlear input impedance and auditory sensitivity [6,7,24,45]. These effects provide a physiologic rationale for considering MEP as a clinically accessible reference variable through which neural, vascular, inflammatory, mechanical, and autonomic influences may become observable.

Within this conceptual model, conditions traditionally described as ETD, pressure-related vertigo, or otherwise unexplained auditory fluctuation may be interpreted as possible expressions of pressure-regulatory instability. However, the phenotypic patterns described below should be understood as hypothesis-generating constructs for future empirical testing, not as definitive disease categories, validated diagnostic phenotypes, or treatment-directing classifications.

The following subsections summarize representative axis-dominant and coupled patterns. Their purpose is to illustrate how the NVMIA framework may support future mechanism-oriented stratification and longitudinal study design, while preserving the need for prospective validation before clinical application.

### 5.1. Conceptual Principles for Interpreting Phenotypic Patterns

Interpretation of NVMIA-based phenotypic patterns rests on three provisional principles. First, individual symptoms may reflect relative predominance of one regulatory axis, but no symptom should be considered specific to a single domain in isolation. Second, most pressure-mediated presentations likely arise from interactions among multiple axes rather than from isolated mechanisms. Third, recurrent symptom clusters may represent reproducible patterns of axis predominance and coupling, but these patterns remain hypothesis-generating until prospectively validated.

This approach is intended to support mechanistic interpretation of fluctuating symptoms without converting the NVMIA framework into a fixed diagnostic classification. Table 4 summarizes representative patterns as illustrative examples only.

### 5.2. Neuro-Dominant Pattern (N-Axis)

The Neuro-dominant pattern may be conceptualized as a pressure-mediated clinical pattern in which altered neural encoding, central sensory gain modulation, and afferent signal instability appear to play a prominent role in symptom expression. In this configuration, mechanical pressure imbalance may function as a triggering or permissive substrate, whereas neural processing dynamics may shape symptom intensity, perceptual salience, and persistence.

Central adaptive responses to altered peripheral input provide the physiological basis for this phenotype. Following unilateral vestibular loss, experimental models demonstrate rapid asymmetry in vestibular nuclei activity followed by progressive central recalibration and plastic rebalancing across brainstem and cerebellar circuits [46,47]. Beyond the brainstem, vestibular and auditory signals are integrated within distributed cortical and limbic networks involved in perception, spatial cognition, and affective modulation [48]. These mechanisms suggest how peripheral perturbations may evolve into sustained neural-axis dominance through altered gain adjustment and network-level reweighting.

Even in the absence of effusion or overt structural pathology, deviations in MEP alter tympanic membrane compliance, ossicular loading, and cochlear input impedance [6,7,24]. Such mechanical modulation can change the pattern and stability of afferent input reaching central auditory and vestibular pathways. Experimental and modeling studies suggest that altered cochlear loading conditions can influence neural encoding dynamics [49], providing a physiologically plausible pathway linking barophysiologic instability to central gain regulation.

In susceptible individuals, unstable afferent signaling may contribute to maladaptive amplification within auditory pathways, potentially manifesting as tinnitus perception, sound intolerance, or sensory distortion [50]. Concurrent engagement of vestibulo–autonomic circuits can further amplify symptom burden beyond what static middle ear measurements would predict [31]. In this proposed phenotype, symptom severity may reflect neural processing vulnerability rather than the magnitude of measurable mechanical abnormality.

Clinically, a Neuro-dominant pattern may show marked state dependence. Symptoms fluctuate with stress, fatigue, barometric variation, swallowing, or autonomic arousal and may transiently intensify during physiologic challenge despite normal or near-normal audiometric or tympanometric findings. This dynamic behavior may help explain why some affected patients are classified as having idiopathic tinnitus, functional dizziness, or primary central vestibular disorders when pressure-mediated afferent instability is not assessed.

Within the NVMIA framework, the Neuro-dominant pattern is proposed as a provisional Neuro–Mechanical coupling pattern modulated by autonomic tone. Pressure instability may initiate perturbation, whereas neural amplification may contribute chronicity and symptom persistence. Restoration of MEP homeostasis may therefore produce clinical improvement that appears disproportionate to static structural findings, although this relationship requires prospective validation.

This pattern remains provisional and should be tested in longitudinal studies linking neural symptom trajectories with MEP dynamics and response profiles.

### 5.3. Vascular-Dominant Pattern (V-Axis)

The Vascular-dominant pattern may be conceptualized as a regulatory pattern in which cochlear or vestibular symptom expression appears to be strongly influenced by microvascular perfusion dynamics, venous pressure regulation, or metabolic vulnerability of the stria vascularis. Within the NVMIA framework, this phenotype does not assume isolated vascular pathology as a universal initiating lesion. Rather, it describes a proposed system configuration in which perfusion-sensitive processes may exert disproportionate influence within a mechanically mediated pressure environment.

The cochlea depends on tightly regulated ionic homeostasis and preservation of the endocochlear potential, sustained by the specialized microvascular architecture of the stria vascularis [21]. Even modest perturbations in perfusion pressure or metabolic buffering capacity may destabilize electrochemical gradients and cochlear amplification efficiency. Contemporary analyses of cochlear microcirculation further emphasize the conditional vulnerability of strial function to systemic and local vascular modulation [22]. Such vulnerability may manifest clinically as fluctuating auditory thresholds or sensory instability in the absence of fixed structural degeneration.

Pulsatile tinnitus provides a clinically recognized example of vascular acoustic coupling. Venous turbulence, jugular bulb asymmetry, or impaired venous drainage can generate hemodynamic signals that may be transmitted to the cochlea [51,52]. However, the perceptibility of these signals depends not solely on vascular flow characteristics, but also on the transfer properties of the middle–inner ear interface.

Classical acoustic investigations demonstrate that pressure-dependent changes in middle ear impedance can alter mechanical transmission characteristics [6,7]. Variations in MEP may therefore modulate the threshold at which vascular acoustic energy becomes perceptible. In this configuration, vascular-dominant phenotypes may therefore be interpreted as dynamic Vascular–Mechanical coupling within a reciprocally regulated system rather than from isolated vascular lesions.

Perfusion-sensitive dizziness or auditory fluctuation may similarly emerge from transient alterations in venous return, systemic hemodynamics, or metabolic reserve. These instabilities may be state-dependent and potentially reversible, distinguishing them conceptually from progressive degenerative pathology. Recognition of this regulatory distinction may help reduce misclassification of perfusion-modulated instability as fixed inner ear disease.

Within precision otology, identification of the Vascular-dominant pattern would depend on detecting relative vascular predominance within a pressure-centered regulatory network.

This pattern remains provisional and should be tested in studies linking vascular sensitivity, MEP variability, and symptom trajectories.

### 5.4. Mechanical-Dominant Pattern (M-Axis)

The Mechanical-dominant pattern may be conceptualized as a regulatory pattern in which instability of MEP-dependent mechanical conditions appears to play a prominent role in clinical expression. Within the NVMIA framework, this phenotype does not imply that mechanical disturbance constitutes the universal origin of pathology. Rather, it reflects a proposed system configuration in which pressure-mediated transmission properties serve as a structural reference plane through which symptom expression may become clinically interpretable.

Patients with a possible Mechanical-dominant pattern may report aural fullness, pressure sensation, autophony, fluctuating conductive or mixed hearing changes, pressure-related dizziness, or non-rotatory vertigo, sometimes in the absence of overt effusion or fixed structural abnormality. These manifestations may arise from dynamic impedance variability and boundary-level mechanical modulation rather than intrinsic cochlear degeneration alone.

Foundational investigations have shown that deviations in MEP are associated with measurable conductive threshold shifts and altered tympanic membrane mechanics [24,53]. Subsequent acoustic analyses demonstrated that pressure alterations can reshape middle ear transfer characteristics and modify ossicular boundary conditions [6,7]. Together, these findings support the interpretation that frequency-dependent acoustic transmission may reflect dynamically shifting impedance states rather than static structural pathology alone.

Beyond threshold correlations, the Mechanical axis is supported by acoustic immittance theory and structural modeling frameworks. Acoustic reflectance and absorbance analyses help formalize how variations in tympanic membrane compliance and ossicular coupling may reshape frequency-specific transmission [38]. Modeling and experimental investigations further suggest that sustained pressure differentials can alter effective system stiffness and impedance behavior within physiologic ranges [54,55]. Complementary studies of fluid-mediated mass loading reveal distinct acoustic patterns associated with altered effective mass [8]. Collectively, these findings support pressure-driven acoustic variability as a structurally grounded and dynamically regulated component of the proposed Mechanical axis.

Adaptive pressure equilibration depends on coordinated ET opening mechanics, primarily mediated by tensor veli palatini muscle activity. Disruption of this physiologic opening mechanism may compromise middle ear ventilation and predispose to persistent pressure disequilibrium, even in the absence of fixed structural obstruction [56]. Within Mechanical-dominant configurations, impaired equilibration—rather than absolute pressure magnitude alone—may constitute a major destabilizing factor.

Within the Mechanical axis, two interrelated but conceptually distinct mechanical layers may be considered: impedance-dominant configurations within the middle ear cavity and interface-dominant configurations at the middle–inner ear boundary. This distinction is intended to clarify how pressure-mediated mechanical instability may produce heterogeneous auditory and vestibular manifestations while remaining within a shared pressure-centered regulatory framework.

This pattern remains provisional and should be tested by assessing impedance variability, MEP dynamics, and response profiles over time.

#### 5.4.1. Impedance-Dominant Configurations

Impedance-dominant configurations may be conceptualized as cavity-level mechanical patterns arising from alterations in effective stiffness or mass of the tympano-ossicular system. These changes can produce frequency-dependent transmission patterns that may help differentiate pressure-related mechanical states within the broader Mechanical axis.

##### Stiffness-Dominant Configuration

A stiffness-dominant configuration may occur during negative middle ear pressure or increased membrane tension, reducing compliance of the tympano-ossicular system. This state tends to attenuate low-frequency transmission while relatively preserving higher frequencies. Experimental models of negative MEP have demonstrated low-frequency conductive shifts and altered tympanic membrane mechanics [24,53]. Clinically, such states may be associated with low-frequency hearing fluctuation, pressure sensation, and potentially reversible auditory instability.

##### Mass-Dominant Configuration

A mass-dominant configuration may emerge when inertial loading becomes a major impedance determinant, most commonly in the presence of middle ear fluid or increased effective mass within the tympanic cavity. In contrast to stiffness-dominant states, mass loading tends to attenuate higher-frequency transmission. Experimental data indicate that middle ear fluid can reduce high-frequency acoustic energy transfer and alter ossicular motion patterns [8]. Wideband reflectance and absorbance analyses further help characterize frequency-dependent signatures that may distinguish mass- and stiffness-dominant mechanical patterns [38], providing in vivo correlates of load-dependent impedance behavior.

Together, these impedance configurations describe the cavity-level mechanical layer of the proposed M-axis. They are intended as conceptual mechanical subpatterns rather than validated diagnostic subtypes.

#### 5.4.2. Interface-Dominant Configuration (Window Coupling)

Beyond cavity-level impedance regulation, mechanical effects may also emerge at the middle–inner ear interface. Interface-dominant configurations are proposed as mechanical states in which altered boundary conditions at the oval or round windows influence intracochlear energy distribution, even in the absence of overt cavity-level impedance instability.

Numerical and experimental studies suggest that mechanical boundary conditions at the window interface can influence round window vibration behavior and intracochlear energy transfer dynamics [57]. These observations support the concept that pressure-mediated changes may alter cochlear input through redistribution of mechanical energy rather than through global changes in middle ear impedance alone. Such interface-level modulation may occur even when conventional tympanometric classification or pure-tone thresholds remain within normal or near-normal limits.

Within this framework, interface-dominant configurations are interpreted as boundary-level energy redistribution rather than isolated cavity-level stiffness or mass effects. This distinction extends the Mechanical axis beyond tympano-ossicular loading and provides a layered interpretation of pressure-related transmission behavior. In the proposed NVMIA architecture, the interface layer serves as a mechanistic bridge linking measurable pressure conditions to cochlear input modulation.

Importantly, interface-dominant configurations remain mechanically expressed but may be shaped by interacting regulatory influences. Inflammatory changes may alter tissue compliance at pressure interfaces; autonomic modulation may influence pressure equilibration dynamics; neural mechanisms may affect reflexive adaptation; and vascular conditions may influence tolerance to pressure-related loading. Accordingly, mechanical expression should not be interpreted as evidence of isolated mechanical causation.

Clinically, this proposed pattern may demonstrate state dependence and partial reversibility. Symptoms may fluctuate with changes in MEP homeostasis despite limited structural findings. Failure to recognize such pressure-sensitive interface behavior may contribute to misclassification as irreversible inner-ear disease or primary neural dysfunction.

At present, interface-dominant configurations should be regarded as hypothesis-generating mechanical subpatterns rather than validated diagnostic entities. Future studies are needed to determine whether pressure-sensitive interface behavior can be reproducibly identified and whether such patterns predict symptom trajectory, physiologic reversibility, or treatment response.

### 5.5. Inflammatory-Dominant Pattern (I-Axis)

The Inflammatory-dominant pattern may be conceptualized as a regulatory pattern in which mucosal immune activation and epithelial inflammatory processes appear to play a prominent role in destabilizing MEP regulation. Within the NVMIA framework, inflammation is not treated as a purely local irritative phenomenon but as a dynamic regulatory modifier that may alter the mechanical properties of the ET interface and reshape the pressure-transmission environment.

Patients with a possible Inflammatory-dominant pattern may report nasopharyngeal pressure, blockage sensation, chronic mucus or postnasal drip, throat discomfort, and pressure-related aural symptoms that fluctuate with inflammatory burden. Upper airway inflammatory conditions, including allergic rhinitis and laryngopharyngeal reflux (LPR), have been associated with mucosal edema and epithelial hypersensitivity in the pharyngolaryngeal region [58,59]. Such inflammatory alterations may extend to the nasopharyngeal ET orifice, modifying epithelial surface characteristics, local compliance, and opening dynamics.

From a biomechanical perspective, inflammation may alter mucosal viscoelastic properties, increase surface adhesion forces between opposing ET walls, and elevate the energetic threshold required for effective tubal opening. Ventilatory failure in this proposed phenotype may therefore reflect state-dependent loss of physiologic compliance rather than fixed anatomical obstruction alone [17]. Structural imaging and endoscopy may appear unremarkable, yet functional opening dynamics remain impaired due to inflammation-mediated reduction in compliance.

Inflammatory-dominant patterns are unlikely to occur in isolation. Reduced mucosal compliance may amplify pressure imbalance within the middle ear, while inflammatory sensory sensitization may interact with autonomic tone to heighten perceptual salience during swallowing, phonation, stress, or reflux episodes. Symptom fluctuation may therefore reflect Inflammatory–Mechanical coupling within a reciprocally regulated pressure system rather than static structural disease alone.

Within precision otology, recognition of an Inflammatory-dominant pattern may help contextualize inflammation-mediated modulation of the mechanical reference plane. This pattern suggests that future studies should evaluate whether changes in mucosal status, reflux-related symptom timing, inflammatory burden, ET opening dynamics, and MEP stability are associated with pressure-regulatory improvement. These relationships remain hypothesis-generating and should not be interpreted as a treatment-directing framework or validated therapeutic algorithm.

This pattern remains provisional and should be tested through longitudinal assessment of mucosal status, MEP stability, and symptom fluctuation.

### 5.6. Autonomic-Dominant Pattern (A-Axis)

The Autonomic-dominant pattern may be conceptualized as a regulatory pattern in which autonomic tone—particularly shifts in vagal–sympathetic balance—appears to strongly modulate symptom expression within the pressure-regulated system. Within the NVMIA framework, the Autonomic axis is proposed as a dynamic state-control layer that may influence the mechanical reference plane of MEP regulation rather than constituting fixed structural pathology.

Patients with a possible Autonomic-dominant pattern may report episodic dizziness, non-rotatory vertigo, presyncope, nausea, pressure fluctuation, globus sensation, or symptom exacerbation during stress, fatigue, postural change, swallowing, or gastrointestinal activity. Objective otologic testing may be intermittently normal, reflecting the state-dependent and reversible nature of the dominant regulatory disturbance.

Mechanistically, autonomic tone may influence ET function and pressure regulation through several converging pathways. Vagal activity may modulate pharyngeal muscle coordination and tubal opening timing, while sympathetic activation may influence mucosal vascular capacitance, secretion dynamics, and sensory gain. Experimental and clinical investigations support the plausibility that pressure-mediated symptoms may, in some cases, emerge from autonomic–mechanical reciprocity rather than primary labyrinthine degeneration alone [31]. Susceptibility to motion-induced dizziness further illustrates the interaction between vestibular processing and autonomic responsiveness [16].

In this configuration, MEP may serve as a dynamic barophysiologic interface: autonomic shifts may alter tubal opening behavior, mucosal vascular tone, and perceptual gain, thereby reshaping the effective mechanical boundary conditions through which pressure signals are expressed. Autonomic-dominant patterns are unlikely to exist in isolation. Autonomic predominance may amplify inflammatory signaling through neuroimmune pathways, alter vascular responsiveness, and enhance central perceptual gain. Symptom expression may therefore reflect a regulatory sequence in which autonomic control networks modulate pressure-sensitive mechanical expression.

Within precision otology, recognition of autonomic predominance may help preserve the conceptual possibility of reversibility. This pattern suggests that autonomic state markers may be relevant variables for future studies of pressure-regulatory instability. However, these intervention-related implications remain provisional and require validation through longitudinal assessment and response-pattern studies.

Failure to consider this regulatory configuration may contribute to misclassification as primary anxiety, functional dizziness, or irreversible inner ear disease. Conversely, over-attribution to autonomic mechanisms should also be avoided unless supported by symptom timing, physiologic context, and objective reassessment.

This pattern remains provisional and should be tested by linking autonomic state markers with MEP dynamics and symptom trajectories.

### 5.7. Mixed and Coupled Patterns: Dynamic Regulatory Interactions

In routine clinical practice, barophysiologic disorders may not conform to a single dominant NVMIA axis. Many clinical presentations are more appropriately conceptualized as mixed or coupled patterns in which two or more regulatory domains interact dynamically over time. Symptom predominance may shift with posture, stress, swallowing frequency, inflammatory burden, sleep state, or environmental pressure variation. Within the proposed framework, such variability may be interpreted, not merely as diagnostic inconsistency, but as a potential feature of reciprocally coupled pressure-regulatory physiology.

Across these mixed states, MEP instability may serve as a shared mechanical reference plane through which interacting regulatory influences become clinically observable. Even modest changes in impedance or pressure equilibrium may alter tympanic membrane–ossicular loading and cochlear input characteristics, thereby influencing afferent stability and symptom perception. When combined with altered neural gain, autonomic modulation, inflammatory sensitization, or vascular susceptibility, these perturbations may generate symptom patterns that appear disproportionate to static structural findings [31].

Within the NVMIA framework, mixed patterns may be interpreted as dynamic regulatory configurations rather than fixed diagnostic entities. A clinically observable presentation may therefore reflect a dominant regulatory layer together with one or more secondary amplification pathways. In this context, symptom fluctuation is considered a potentially informative physiologic feature rather than evidence of purely functional symptoms or irreversible pathology.

Importantly, these coupled patterns should not be assumed to imply fixed structural disease. Rather, they may represent partially reversible interaction among multiple regulatory domains within a dynamically regulated pressure system. Recognition of such patterns may help reduce premature attribution to irreversible inner-ear disease while supporting focused reassessment of the most prominent regulatory contributor and its secondary modulators.

Understanding mixed patterns in this manner may inform future studies of longitudinal symptom tracking, repeated physiologic measurement, and cross-axis interaction. Such studies should determine whether suspected dominant regulatory layers and secondary amplification pathways can be identified reproducibly over time and whether they are associated with symptom trajectory, pressure stability, or response profiles.

At this stage, mixed and coupled patterns should be interpreted as hypothesis-generating conceptual constructs intended to support future mechanism-oriented phenotyping rather than as validated clinical classifications or treatment-directing frameworks.

### 5.8. Potential Research Utility of NVMIA-Based Patterning

NVMIA-based patterning may provide a provisional structure for organizing fluctuating or ambiguous barophysiologic presentations according to suspected regulatory predominance and cross-axis coupling. Its primary utility at this stage is conceptual and research-oriented: it may help generate testable hypotheses about why similar symptom labels show divergent trajectories and why apparently normal static findings may coexist with clinically meaningful symptom variability.

Conventional evaluation often relies on single-point structural testing, whereas pressure-mediated disorders may fluctuate across physiologic state, inflammatory burden, autonomic tone, and environmental context. Within the NVMIA framework, such variability is interpreted not as diagnostic inconsistency alone, but as a potential signal for longitudinal assessment and future stratification studies.

Accordingly, NVMIA-based patterning should not be regarded as a validated clinical decision tool or treatment-directing classification. Its proposed value lies in guiding future research designs that integrate structural metrics, pressure-related physiologic variability, and patient-reported symptom dynamics to test whether regulatory patterns can improve phenotypic stratification, outcome prediction, and mechanism-aligned intervention studies.

### 5.9. Conceptual Summary

NVMIA-based patterning provides a conceptual bridge between pressure-centered mechanistic interpretation and future studies of mechanism-aligned assessment. In this framework, symptom variability is interpreted as a potential signal of dynamic regulatory interaction rather than as diagnostic inconsistency alone.

## 6. Provisional Research Implications for Mechanism-Oriented Intervention Within the NVMIA Framework

Because the NVMIA framework is proposed as a conceptual and hypothesis-generating architecture, its intervention-related implications should be interpreted cautiously. The framework does not establish a validated treatment algorithm, procedural hierarchy, or clinical decision pathway. Rather, it suggests that future studies may evaluate whether suspected regulatory predominance is associated with distinct pressure-related response patterns.

Within this provisional model, the Mechanical domain remains a clinically accessible reference plane because MEP and impedance measures provide measurable outputs of pressure regulation. However, future evaluation should not assume that pressure-mediated disorders require mechanical correction as the initial or universal approach. Neural gain instability, vascular susceptibility, inflammatory modulation, impedance instability, and autonomic state dependence are discussed here as candidate domains for research rather than as prescriptive treatment targets.

**Figure 3:**
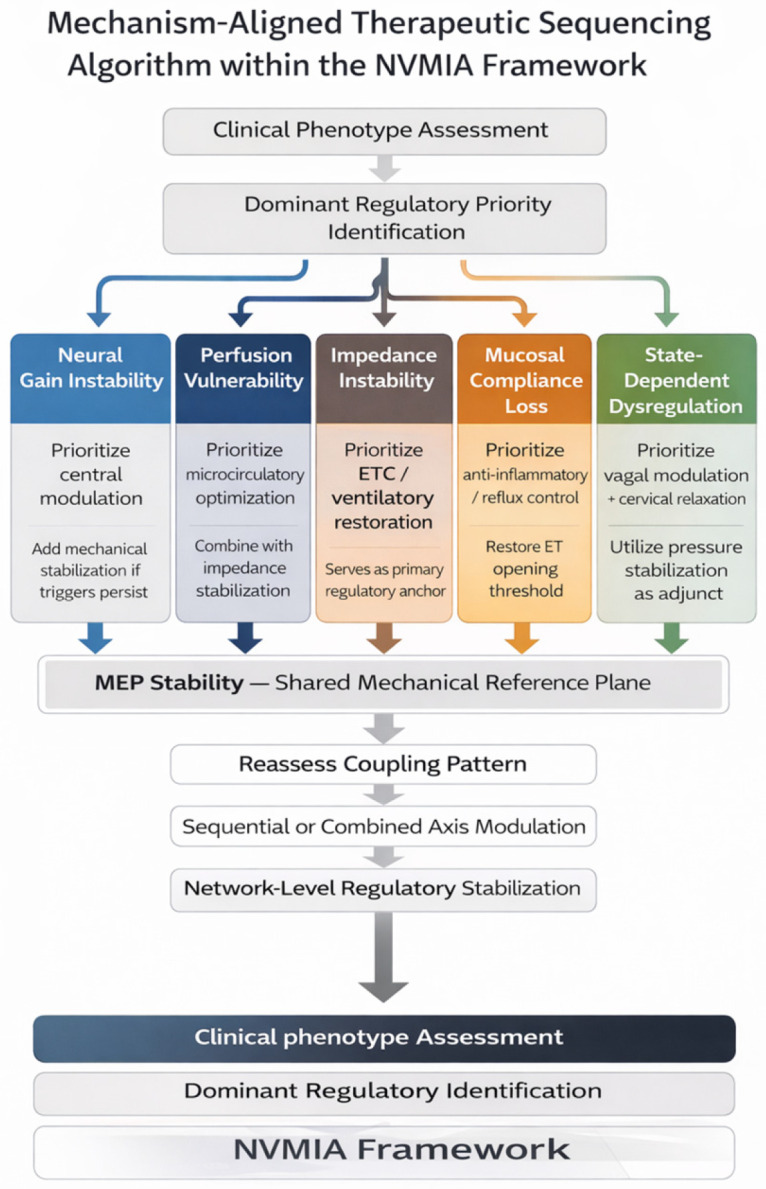
Conceptual framework for future evaluation of mechanism-oriented intervention studies within the Neuro–Vascular–Mechanical–Inflammatory–Autonomic (NVMIA) framework. The figure illustrates how clinical evaluation, provisional identification of suspected regulatory predominance, and reassessment of pressure-related outcomes may be organized for future empirical testing. Middle ear pressure (MEP) is interpreted as a shared mechanical reference variable for evaluating pressure-regulatory stability. The sequence is intended as a hypothesis-generating research framework for studying possible relationships among suspected dominant domains, cross-domain coupling, and response patterns, and requires prospective validation before any clinical application.

The following subsections therefore present illustrative intervention-related research implications rather than clinical recommendations. They are intended to support future study design and hypothesis generation, not to define validated NVMIA-directed treatment pathways. The proposed mechanism-aligned sequencing framework for future evaluation is illustrated in Figure 3.

### 6.1. Provisional Axis-Specific Research Considerations

Within the proposed NVMIA framework, axis-specific considerations are presented as provisional research concepts rather than as treatment priorities or a validated sequencing strategy. Their purpose is to generate testable hypotheses regarding whether suspected regulatory predominance is associated with distinct pressure-related response patterns. Neural gain instability, perfusion vulnerability, impedance instability, mucosal compliance alteration, and autonomic state imbalance are therefore discussed as candidate domains for future evaluation rather than as prescriptive treatment targets.

Across these provisional patterns, MEP may function as a clinically accessible reference variable through which pressure-related physiologic change can be monitored in future studies. Reassessment should be understood as a research-oriented process involving objective pressure-related measures, patient-reported outcomes, symptom timing, and possible persistence of cross-axis coupling, rather than as a validated clinical decision pathway.

#### 6.1.1. Neural Gain Instability (Neuro-Dominant Pattern)

When maladaptive central gain, altered neural encoding, or afferent signal volatility appears to predominate, this pattern may support future evaluation of sensory amplification as a response-related variable rather than assuming immediate structural correction alone explains symptom change. Even modest deviations in MEP, in the absence of effusion or overt pathology, may destabilize cochlear input impedance and alter afferent firing patterns [6,7,24]. In susceptible individuals, such peripheral instability may facilitate central gain enhancement, contributing to tinnitus perception, auditory distortion, or pressure-associated dizziness [50].

For future empirical evaluation, studies should examine whether measures of neural amplification, symptom fluctuation, and pressure-related afferent instability are associated with patient-reported outcomes and response profiles. When pressure instability persists as a recurrent trigger, future studies should evaluate whether pressure stabilization modifies neural amplification using both symptom-based and objective pressure-regulatory measures.

#### 6.1.2. Perfusion Vulnerability (Vascular-Dominant Pattern)

In vascular-dominant patterns, symptom fluctuation may reflect perfusion sensitivity of cochlear or vestibular microcirculation rather than fixed structural degeneration alone [21,52]. Because MEP instability may modify cochlear boundary conditions and acoustic transmission across the middle–inner ear interface [8,38], perfusion vulnerability may be amplified within a pressure-sensitive mechanical environment.

For future empirical evaluation, studies should examine whether perfusion-related markers, metabolic reserve indicators, venous outflow characteristics, impedance measures, and MEP variability are associated with pressure-sensitive auditory or vestibular fluctuation. The key question is whether physiologic reserve, pressure variability, and symptom trajectories show reproducible longitudinal relationships.

#### 6.1.3. Impedance Instability (Mechanical-Dominant Pattern)

Mechanical-dominant patterns may be suspected when instability of MEP and impedance mismatch at the tympanic membrane–ossicular interface appears to represent the major measurable disturbance. Even modest deviations in pressure can alter tympanic membrane stiffness, ossicular loading, cochlear impedance, and round- and oval-window mechanics [24,45], thereby reshaping auditory transmission and vestibular boundary conditions.

For future empirical evaluation, studies should examine whether pressure-response profiles, ventilatory function measures, impedance changes, repeated MEP profiles, and pressure-oriented physiologic approaches are associated with improved pressure-regulatory stability and patient-reported outcomes. In selected clinical contexts, Eustachian tube catheterization may be considered a candidate physiology-oriented approach for study because it directly targets tubal opening mechanics and MEP regulation. A previously reported case of ground-level alternobaric vertigo associated with ETD provides an illustrative clinical observation in which pressure-related vestibular symptoms improved after restoration of Eustachian tube function [19]. This observation is interpreted as hypothesis-generating rather than as validation of a generalized therapeutic algorithm.

Within this conceptual model, the relevant research question is not merely whether a static pressure value can be transiently normalized, but whether adaptive pressure regulation can be evaluated across physiologic contexts. Potential secondary associations with neural gain, perfusion sensitivity, or autonomic amplification remain subjects for future validation.

#### 6.1.4. Mucosal Compliance Loss (Inflammatory-Dominant Pattern)

Inflammatory-dominant patterns may reflect impaired ET compliance due to mucosal edema, immune activation, allergic inflammation, or reflux-mediated irritation [4,5,58]. Inflammatory processes may increase surface adhesion forces and elevate the energetic threshold for tubal opening, destabilizing pressure equilibration despite preserved static anatomy.

For future empirical evaluation, studies should examine whether mucosal status, allergic disease activity, reflux-related symptom timing, inflammatory markers, ET opening dynamics, and MEP stability are associated with symptom fluctuation and pressure-regulatory instability. The key question is whether changes in inflammatory burden and mucosal compliance correspond to reproducible changes in tubal opening behavior, longitudinal MEP profiles, and patient-reported outcomes.

#### 6.1.5. State-Dependent Dysregulation (Autonomic-Dominant Pattern)

Autonomic-dominant presentations may be suspected when symptom fluctuation is associated with stress, posture, swallowing, exertion, or gastrointestinal activity [16,31]. Altered vagal–sympathetic balance may influence ET opening coordination, mucosal vascular capacitance, and MEP stability.

For future empirical evaluation, this pattern suggests that studies should examine whether autonomic state markers, stress-related symptom timing, respiratory pattern, gastrointestinal symptom context, and repeated pressure-related measures are associated with state-triggered symptom amplification. Longitudinal assessment should evaluate relationships among physiologic context, objective pressure-related measures, patient-reported outcomes, and residual cross-axis coupling.

### 6.2. Coupled/Multiaxial Instability: Network-Level Regulatory Dynamics

Barophysiologic disorders may present as multiaxial regulatory patterns in which two or more NVMIA domains interact dynamically across physiologic context and time. Within these configurations, MEP instability may serve as a shared mechanical reference plane through which network-level dysregulation becomes clinically interpretable. Impedance shifts can modify middle ear boundary conditions and acoustic transfer characteristics [8], producing variability in afferent input that may be further shaped by neural plasticity and autonomic modulation [31].

As summarized in Table 5, coupled patterns are proposed as provisional regulatory constructs rather than fixed diagnostic categories. Their purpose is to organize possible relationships among suspected dominant-axis activity, secondary amplification pathways, state dependence, and response variability. Within this conceptual framework, future studies may evaluate whether identifying the most clinically influential axis and reassessing cross-axis coupling improves interpretation of fluctuating pressure-mediated symptoms.

**Table 5:** Illustrative coupled regulatory patterns within the NVMIA framework. This table summarizes representative mixed presentations that may be conceptually interpreted as dynamic interaction states rather than diagnostic inconsistencies. These patterns are intended as hypothesis-generating aids for future empirical testing rather than clinical guidance tools.

Interaction Pattern	Proposed Interaction Driver	Candidate Evaluation Focus	Hypothesized Interaction Logic	Potential Research Endpoint
Mechanical–Autonomic Coupling	Autonomic arousal interacting with pressure variability	Autonomic state measures, contextual triggers, and repeated pressure measures	Autonomic arousal, MEP variability, and symptom amplification may interact longitudinally	Longitudinal association among autonomic state, MEP variability, and symptom fluctuation
Neuro–Mechanical Coupling	Pressure-related afferent instability interacting with central gain modulation	Pressure-response profiles, sensory amplification measures, tinnitus or dizziness fluctuation, and neural gain markers	MEP instability may modify afferent input and contribute to perceptual amplification	Association between pressure variability and sensory amplification over time
Autonomic–Inflammatory Coupling	Autonomic state interacting with mucosal variability and inflammatory burden	Autonomic markers, mucosal status, reflux-related timing, inflammatory indicators, and symptom diaries	Autonomic tone and inflammatory burden may jointly alter ET opening thresholds and pressure variability	Longitudinal association between autonomic–inflammatory state and pressure-regulatory instability
Coupled/Multiaxial Instability	Dynamic interaction among multiple regulatory domains	Repeated MEP measures, PROMs, contextual triggers, suspected dominant-domain markers, and cross-domain coupling indicators	Dominant and secondary regulatory influences may shift across physiologic context and time	Patterns of network-level regulatory variability and reproducibility of suspected coupling states

Possible sequential or combined axis-directed approaches should therefore be understood as hypothesis-generating research concepts rather than validated treatment protocols. Such approaches would require prospective validation using symptom timing, objective pressure-related measures, contextual triggers, patient-reported outcomes, and longitudinal response patterns.

Failure to consider multiaxial interaction may contribute to incomplete interpretation of symptom variability, whereas over-interpretation of axis coupling should also be avoided unless supported by physiologic context and repeated assessment. In this sense, the network-regulatory model is intended to preserve the conceptual possibility of reversibility while guiding future studies of mechanism-aligned assessment and intervention.

Table 6 summarizes illustrative single-axis regulatory considerations organized around MEP-centered physiologic variability, while Table 5 summarizes representative coupled-domain patterns for future empirical evaluation. Both tables are intended as conceptual aids for future empirical testing rather than clinical guidance tools.

**Table 6:** Illustrative single-axis regulatory considerations within the NVMIA framework. This table summarizes how pressure-related regulatory patterns may be conceptually organized according to suspected dominant physiologic drivers with middle ear pressure (MEP) serving as a shared reference variable for future reassessment. These considerations are intended as hypothesis-generating aids for future empirical testing rather than clinical guidance tools.

Illustrative Regulatory Pattern	Primary Physiologic Focus	Candidate Study Focus	Hypothesized System-Level Observation
Neural Gain Instability	Central auditory/vestibular gain	Neural gain markers, sensory amplification measures, and symptom-response tracking	Association between pressure-related afferent instability and perceptual amplification
Perfusion Vulnerability	Cochlear microcirculation & venous flow	Perfusion-related markers, pressure variability, and auditory fluctuation tracking	Association between vascular susceptibility and pressure-sensitive auditory or vestibular variability
Impedance Instability	Middle ear pressure (MEP) regulation	Pressure-response assessment, impedance measures, ventilatory function, and repeated MEP profiles	Changes in transmission efficiency and pressure-regulatory stability
Mucosal Compliance Loss	Eustachian tube mucosal and inflammatory state	Mucosal status, reflux-related symptom timing, inflammatory markers, and MEP stability	Association between mucosal status, ET opening dynamics, and MEP stability
State-Dependent Dysregulation	Autonomic tone and contextual physiologic state	Autonomic state markers, contextual triggers, symptom timing, and repeated pressure measures	Association between autonomic state and pressure-related symptom variability

### 6.3. Provisional Intervention-Related Reasoning and Future Precision Otology

Within the proposed NVMIA framework, intervention-related reasoning is presented as provisional and hypothesis-generating rather than as a validated treatment sequence. Future studies may evaluate whether suspected regulatory predominance and cross-axis coupling improve interpretation of pressure-mediated symptom variability and response heterogeneity.

In this model, MEP is interpreted as a measurable clinical reference plane through which multisystem instability may become more physiologically interpretable and suitable for longitudinal reassessment. A provisional research logic may therefore involve identifying the suspected dominant axis, assessing pressure-related stability, reassessing cross-axis interaction over time, and evaluating whether adjunctive modulation is associated with improved regulatory stability.

Response patterns should not be interpreted solely as transient symptom suppression, but may be studied in relation to pressure stability, reproducibility of response, patient-reported outcomes, and preservation of physiologic reversibility. This regulation-centered perspective is intended to support future empirical testing of precision otology concepts rather than to establish a validated clinical treatment pathway.

## 7. Future Directions: Validation, Computational Translation, and Precision Otology

The NVMIA framework is proposed as a pressure-centered conceptual architecture for organizing ETD and related barophysiologic disorders. Its translational value will depend on whether the proposed regulatory axes, dynamic MEP-related measures, and response patterns can be empirically tested in prospective studies.

Future work should prioritize reproducible measurement of MEP variability, interaural pressure asymmetry, pressure-response patterns after physiologic challenge, and their relationship to patient-reported outcomes. Such studies will be necessary before NVMIA-based patterning can be used as a formal diagnostic classification or treatment-directing framework.

### 7.1. Empirical Validation of NVMIA-Based Patterns

The first priority is to test whether the proposed NVMIA-based patterns can be identified reproducibly. In this manuscript, Neural-, Vascular-, Mechanical-, Inflammatory-, Autonomic-, and mixed-axis patterns are presented as hypothesis-generating constructs rather than established clinical categories. Future studies should determine whether these patterns can be identified reproducibly using combinations of MEP measurement, tympanometry, wideband acoustic immittance, tubal function testing, vestibular assessment, mucosal evaluation, autonomic markers, and patient-reported outcomes.

Both cross-sectional and longitudinal designs will be required. Cross-sectional studies may evaluate whether suspected axis-dominance patterns correspond to distinct physiologic or symptom profiles. Longitudinal studies should test whether these patterns remain stable, shift over time, or predict response trajectories. Particular attention should be given to discordance between objective pressure stabilization and persistent symptoms, as this may provide testable evidence of secondary Neural or Autonomic amplification.

### 7.2. Dynamic MEP-Based Outcome Studies

A second priority is the development of dynamic MEP-based outcome studies. Single-timepoint tympanometry provides useful baseline information but may not capture the temporal instability, contextual sensitivity, and interaural asymmetry that characterize pressure-mediated disorders. Future studies should therefore evaluate repeated MEP measurements, symptom-linked pressure fluctuation, pressure response after swallowing or yawning, and longitudinal patterns of pressure stabilization.

MEP should not be treated as a validated surrogate biomarker on the basis of static measurement alone. Rather, dynamic MEP profiles may represent candidate physiologic signals for future validation. Potential endpoints include MEP variability, interaural asymmetry, pressure-response patterns, wideband immittance changes, symptom fluctuation, dizziness episodes, hearing threshold variability, and response trajectories.

### 7.3. Future Computational Integration

Future computational models in otology may benefit from incorporating physiologically meaningful variables rather than relying solely on downstream phenotypic outputs such as audiometric profiles, eye movement patterns, imaging features, or symptom clusters. Within the NVMIA framework, MEP dynamics, impedance variability, mucosal status, vascular susceptibility, neural adaptation, and autonomic state may represent candidate input domains for future interpretable models. Prior perspective work has similarly emphasized that middle ear pressure physiology may warrant consideration in future AI-driven vertigo research [60].

However, computational application of NVMIA remains preliminary. The framework is not proposed here as an AI-based diagnostic system or clinical decision tool. Its potential computational role is limited to future hypothesis testing, longitudinal pattern recognition, and evaluation of whether physiologically grounded variables improve interpretability after prospective validation.

### 7.4. Multicenter and Digital Health Directions

A practical next step is the development of multicenter observational protocols capable of capturing real-world pressure-regulatory variability. Such studies could combine repeated tympanometry, wideband acoustic immittance, symptom diaries, ETD-specific PROMs, dizziness or pressure-intolerance scales, and longitudinal response monitoring.

Digital health tools may support longitudinal symptom tracking and time-stamped correlation among symptoms, contextual triggers, and objective pressure-related measures. These approaches may also inform future PROM development, particularly for pressure-sensitive vestibular, autonomic, reflux-associated, and context-dependent symptoms.

Overall, the immediate role of the NVMIA framework is conceptual and translational: to organize existing physiologic knowledge, identify testable relationships, and provide a structured basis for future validation studies in precision otology.

## 8. Discussion

Contemporary approaches to ETD and related otologic disorders remain largely anchored in structure-based diagnosis and symptom classification. While clinically practical, such approaches may be limited in conditions characterized by fluctuating physiology, multisystem interaction, and state-dependent reversibility. The proposed NVMIA framework addresses this conceptual gap by organizing pressure-mediated symptoms around MEP as a measurable physiologic variable within a reciprocally coupled multiaxial system.

Rather than attributing symptom patterns to isolated structural abnormalities or fixed disease entities, the framework interprets clinical manifestations as possible expressions of pressure-regulatory states. This interpretation remains provisional, but it may provide a useful structure for future studies examining mechanistic predominance, cross-axis coupling, and regulatory instability.

### 8.1. Conceptual Advancement: From Descriptive Labels to Regulatory Architecture

Traditional diagnostic categories remain indispensable for communication and classification. However, in conditions characterized by state dependence, physiologic fluctuation, and multisystem coupling, categorical labels may provide limited explanatory resolution [1]. Patients sharing a diagnostic label may exhibit heterogeneous symptom constellations whose variability cannot be fully accounted for by static anatomy alone [4,5].

The NVMIA framework reorganizes interpretation by relating symptom expression to proposed regulatory configurations rather than to descriptive labels alone. The conceptual shift is therefore not from diagnosis to abstraction, but from label-centered reasoning toward regulation-centered stratification.

### 8.2. Mechanistic Coherence and Clinical Implications

Positioning MEP regulation as a clinically accessible reference plane may offer conceptual advantages because MEP is measurable, dynamically variable, and linked to ET physiology. Within the NVMIA framework, MEP is interpreted as a candidate convergence interface at which mechanical impedance, vascular modulation, neural encoding, mucosal compliance, and autonomic tone may interact.

This positioning may help reinterpret clinical heterogeneity as regulatory configuration rather than diagnostic inconsistency alone. Similar symptom clusters may arise from different suspected axis-dominance patterns, such as neural gain instability, inflammatory modulation, autonomic state dependence, or impedance instability. However, these patterns remain provisional and require prospective validation before they can support formal clinical stratification.

Importantly, the framework does not imply that MEP alone explains otologic symptomatology. Rather, it proposes that pressure regulation may interact with broader physiologic processes to shape perceptual and functional outcomes.

### 8.3. Future Translational and Computational Implications

The NVMIA framework may also provide a structure for future translational studies. Differences in neural gain adaptation, vascular resilience, inflammatory burden, and autonomic state may help explain why patients with comparable structural findings can exhibit divergent clinical trajectories. These relationships should be tested through longitudinal studies incorporating dynamic MEP measures, impedance assessment, patient-reported outcomes, and contextual symptom tracking.

Computational applications should be interpreted cautiously. The NVMIA framework is not proposed as an AI-based diagnostic system or clinical decision tool. Rather, future mechanism-aware models may evaluate whether dynamic MEP profiles, impedance measures, symptom trajectories, and selected physiologic markers improve interpretability after prospective validation. At this stage, computational modeling remains a future research direction rather than an established component of clinical practice.

### 8.4. Limitations

Several limitations warrant consideration.

First, the NVMIA framework remains conceptual and integrative rather than a validated clinical algorithm. Although grounded in established physiologic principles [4,5], prospective validation will be required to evaluate its reproducibility and clinical relevance.

Second, scalable measurement of dynamic MEP variability and ET opening behavior remains technically constrained in routine clinical practice. Standardized protocols for repeated measurement, contextual symptom assessment, and patient-reported outcome integration will be needed before broader application can be considered.

Third, although ETD provides a useful model, generalization to broader domains of precision otology or personalized medicine will require domain-specific adaptation and validation.

Fourth, the five-domain architecture represents a parsimonious selection of proximal physiologic interfaces influencing pressure homeostasis. Systemic, endocrine, metabolic, genetic, or psychosocial influences are not excluded; rather, they are conceptualized as modulatory forces that may operate through one or more core regulatory domains.

Finally, the intervention-related implications described in this review are provisional. They are intended to support hypothesis generation and future study design rather than to define a prescriptive treatment pathway or clinical guideline.

### 8.5. Future Directions

Future investigation should prioritize three complementary pathways: development of standardized metrics for dynamic MEP variability and ET physiology; prospective studies evaluating whether suspected axis-dominance patterns correlate with symptom trajectories and response patterns; and cautious integration of NVMIA-informed physiologic variables into future computational models.

Overall, the NVMIA framework should be understood as a conceptual scaffold for future empirical validation. Its immediate value lies in organizing physiologic complexity, identifying testable relationships, and supporting future precision otology research rather than establishing an immediately applicable diagnostic or therapeutic system.

## 9. Conclusions: From Regulatory Instability to Future Precision Otology

The NVMIA framework proposes a pressure-centered conceptual architecture for interpreting ETD and related pressure-mediated otologic disorders as dynamic regulatory states rather than static structural abnormalities alone. By positioning middle ear pressure within a multiaxial physiologic network, the framework links fluctuating phenotypic patterns to interacting Neural, Vascular, Mechanical, Inflammatory, and Autonomic domains.

This model is not presented as a validated diagnostic or therapeutic algorithm, but as a hypothesis-generating framework for future studies of regulatory patterning, dynamic MEP assessment, patient-reported outcomes, and mechanism-oriented validation. Ultimately, precision otology may advance not by refining diagnostic labels alone, but by identifying, characterizing, and empirically validating measurable pressure-regulatory states.

## Figures and Tables

**Table 2:** Conceptual organization of NVMIA regulatory domains. This table summarizes the proposed relationship between each NVMIA domain, representative physiologic roles, measurable features, and illustrative clinical expressions. It is intended as a conceptual interpretive aid rather than as a validated diagnostic checklist, clinical decision tool, or treatment guide. The listed measurable features and clinical expressions are representative and hypothesis-generating, not exhaustive, threshold-defining, or intended to replace conventional diagnostic evaluation. Prospective validation is required before these patterns can be used for formal clinical stratification.

NVMIA Domain	Primary Mechanistic Role	Representative Biomarkers/Measures	Illustrative Clinical Expressions	Research-Oriented Interpretive Implications
Mechanical	Structural transmission of pressure and compliance regulation via Eustachian tube opening, tympanic membrane mobility, and cochlear window mechanics	Tympanometry (MEP), wideband acoustic immittance, ET opening tests, otoscopic mobility	Aural fullness, conductive or mixed hearing loss, pressure-induced vertigo, barotrauma-related symptoms	Candidate domain for future studies of pressure stabilization, reversibility, and response patterns
Neural	Sensory integration, reflex modulation, and central adaptation to altered pressure input across auditory and vestibular pathways	Vestibular evoked responses, symptom variability, central gain patterns, reflexive swallowing/ET coordination	Vertigo, tinnitus, motion sensitivity, symptom amplification or persistence without structural progression	Supports future evaluation of symptom–test discordance using neural gain and pressure-related variability markers
Vascular	Microvascular perfusion and metabolic vulnerability of cochlear and middle ear structures under pressure stress	Cochlear reserve indicators, fluctuation patterns, delayed SNHL progression, autonomic–vascular tone	Delayed or progressive sensorineural hearing loss, pressure-sensitive auditory fatigue	Supports future evaluation of vascular susceptibility, recovery patterns, and pressure-sensitive auditory fluctuation
Inflammatory	Modulation of mucosal compliance, immune activation, and reflux-associated tissue injury affecting tubal patency	Nasopharyngeal inflammation, allergic markers, LPR/GERD indicators, mucosal edema	Fluctuating ETD, recurrent symptoms, association with rhinitis, sinusitis, reflux	Highlights bidirectional ETD–inflammation loop; suggests future evaluation of inflammation–mechanics coupling
Autonomic	Regulation of tubal opening dynamics, vascular tone, and stress-related modulation of pressure control	Autonomic symptoms (nausea, diaphoresis), stress reactivity, positional or state-dependent variability	Nausea, vomiting, sweating, pallor, cardiovascular instability accompanying pressure events	Supports interpretation of state-dependent symptom fluctuation; supports future study of autonomic state dependence in pressure-mediated symptom variability

**Table 3:** Conceptual interpretation of representative NVMIA-related clinical patterns. This table provides illustrative examples of how selected otologic and multisystem clinical expressions may be interpreted in relation to possible dominant and coupled NVMIA domains. Domain assignments are intended to be state-dependent, context-sensitive, and hypothesis-generating rather than fixed etiologic labels. The table is intended as a conceptual aid for future empirical testing and should not be interpreted as a validated diagnostic classification, clinical decision tool, or treatment guide. Patterns may shift across visits and should not be interpreted as fixed patient subtypes.

Clinical Pattern	Primary NVMIA Domain	Coupled Domains	Mechanistic Interpretation
Tinnitus fluctuation	N	M; A	Sensory gain influenced by pressure and autonomic factors
Hyperacusis/sound hypersensitivity	N	M	Increased sensory gain with altered transmission
Non-rotatory dizziness (“floating”)	N	A	Sensory mismatch with autonomic–vestibular interaction
Imbalance perception	N	V	Vestibular integration sensitive to physiologic variability
Sensory gain amplification	N	A	Central hypersensitivity influenced by autonomic regulation
Pulsatile tinnitus (venous type)	V	N	Venous flow associated with altered cochlear perception
Vascular-type dizziness	V	A	Perfusion-sensitive vestibular instability
Head pressure/cranial fullness	V	M	Altered pressure perception with vascular contribution
Perfusion-sensitive SNHL	V	N; M	Auditory fluctuation associated with microvascular vulnerability
Orthostatic dizziness with aural symptoms	V	A; M	Postural perfusion shifts with pressure-sensitive symptoms
Arrhythmia-associated dizziness	V	A; N	Rhythm variability associated with vestibular symptoms
Reflux-triggered throat discomfort	I	A	Mucosal irritation with autonomic interaction
ET mucosal swelling (“sticky ET”)	I	M	Reduced tubal compliance associated with edema
Chronic mucus sensation/postnasal drip	I	A	Mucosal hyperreactivity with autonomic modulation
Nasopharyngeal blockage/pressure	I	M	Increased ET resistance associated with mucosal changes
Aural fullness	M	A	Pressure-related ear sensation with autonomic modulation
Conductive hearing fluctuation	M	N	Variable middle ear transmission with neural adaptation
Low-frequency hearing fluctuation	M	V	Pressure-associated stiffness with low-frequency attenuation
Barometric vertigo/GLABV	M	N; A	Pressure disequilibrium associated with vestibular interaction
Pressure intolerance (airplane/elevator)	M	A	Impaired equalization with heightened sensitivity
Difficulty equalizing (“cannot pop ear”)	M	I	ET opening inefficiency associated with mucosal state
Vagal dizziness after swallowing	A	M	Swallow–pressure–vagal interaction
Reflux-triggered vertigo	A	I	Vagal activation associated with vestibular symptoms
Stress-induced ear pressure spikes	A	M	Stress-associated autonomic influence on pressure perception
Palpitations with ear pressure	A	V; M	Cardiovascular arousal associated with altered perception of ear pressure
Autonomic–ET “panic-like” episodes	A	N; M	Sensory amplification with pressure-related symptoms
Swallowing-induced transient imbalance	A	N; M	Swallow-associated vestibular–pressure interaction

Abbreviations: N, neural; V, vascular; M, mechanical; I, inflammatory; A, autonomic.

**Table 4:** Illustrative pattern-level interpretation of NVMIA-based phenotypes in barophysiologic disorders. This table summarizes representative, hypothesis-generating patterns of suspected axis predominance and cross-axis coupling. It is intended as a conceptual interpretive aid rather than as a validated diagnostic classification system, clinical decision tool, or treatment guideline. Prospective validation is required before these patterns can be used for formal clinical stratification.

Illustrative Pattern	Possible Predominant Domain	Core Mechanism	Typical Symptoms	Research-Oriented Considerations
Neuro-dominant	Neuro > Mechanical	Pressure-modulated neural gain; afferent instability	Tinnitus; sound sensitivity; non-rotatory dizziness	Stress- and state-dependent; normal static tests; future studies may evaluate MEP stability and neural amplification markers
Mechanical-dominant	Mechanical	TM–ossicular stiffness loading; impedance mismatch	Aural fullness; LF hearing loss; pressure intolerance	Altitude/posture sensitive; Type C or stiffness shift; future studies may evaluate pressure-response profiles
Inflammatory-dominant	Inflammatory > Mechanical	Mucosal edema; reduced ET compliance	Ear pressure; blockage sensation; fluctuating hearing	Inflammation- or reflux-associated variability; future studies may evaluate mucosal status, reflux timing, and MEP stability
Autonomic-dominant	Autonomic > Neuro	Autonomic–vestibular dysregulation	Dizziness; nausea; vagal symptoms	Stress/anxiety triggered; poor test–symptom match; future studies may evaluate autonomic markers and pressure variability
Mixed/Coupled	Multi-axis	Dynamic NVMIA interaction	Multisystem fluctuating symptoms	Context-dependent variability; future studies may evaluate repeated MEP measures, symptom timing, and cross-axis coupling

## Data Availability

No new data were created or analyzed in this study. Data sharing is not applicable to this article.

## Abbreviations

The following abbreviations are used in this manuscript:

AI	Artificial Intelligence
ET	Eustachian Tube
ETC	Eustachian Tube Catheterization
ETD	Eustachian Tube Dysfunction
GLABV	Ground-Level Alternobaric Vertigo
LPR	Laryngopharyngeal Reflux
MEP	Middle Ear Pressure
NVMIA	Neuro–Vascular–Mechanical–Inflammatory–Autonomic
PROMs	Patient-Reported Outcome Measures
SNHL	Sensorineural Hearing Loss
WAI	Wideband Acoustic Immittance
